# Complete Revascularization by Percutaneous Coronary Intervention for Patients With ST‐Segment–Elevation Myocardial Infarction and Multivessel Coronary Artery Disease: An Updated Meta‐Analysis of Randomized Trials

**DOI:** 10.1161/JAHA.119.015263

**Published:** 2020-06-01

**Authors:** Yousif Ahmad, James P. Howard, Ahran Arnold, Megha Prasad, Henry Seligman, Christopher M. Cook, Takayuki Warisawa, Matthew Shun‐Shun, Ziad Ali, Manish A. Parikh, Rasha Al‐Lamee, Sayan Sen, Darrel Francis, Jeffrey W. Moses, Martin B. Leon, Gregg W. Stone, Dimitri Karmpaliotis

**Affiliations:** ^1^ Columbia University Medical Center/New York‐Presbyterian Hospital New York NY; ^2^ National Heart and Lung Institute Imperial College London London United Kingdom; ^3^ Cardiovascular Research Foundation New York NY; ^4^ The Zena and Michael A. Wiener Cardiovascular Institute Icahn School of Medicine at Mount Sinai New York NY

**Keywords:** percutaneous coronary intervention, revascularization, ST‐segment–elevation myocardial infarction, Percutaneous Coronary Intervention, Revascularization, Meta Analysis

## Abstract

**Background:**

For patients with ST‐segment–elevation myocardial infarction (STEMI) and multivessel coronary artery disease, the optimal treatment of the non‐infarct‐related artery has been controversial. This up‐to‐date meta‐analysis focusing on individual clinical end points was performed to further evaluate the benefit of complete revascularization with percutaneous coronary intervention for patients with STEMI and multivessel coronary artery disease.

**Methods and Results:**

We systematically identified all randomized trials comparing complete revascularization with percutaneous coronary intervention to culprit‐only revascularization for multivessel disease in STEMI and performed a random‐effects meta‐analysis. The primary efficacy end point was cardiovascular death analyzed on an intention‐to‐treat basis. Secondary end points included all‐cause mortality, myocardial infarction, and unplanned revascularization. Ten studies (7542 patients) were included: 3664 patients were randomized to complete revascularization and 3878 to culprit‐only revascularization. Across all patients, complete revascularization was superior to culprit‐only revascularization for reduction in the risk of cardiovascular death (relative risk [RR], 0.68; 95% CI, 0.47–0.98; *P*=0.037; I^2^=21.8%) and reduction in the risk of myocardial infarction (RR, 0.65; 95% CI, 0.54–0.79; *P*<0.0001; I^2^=0.0%). Complete revascularization also significantly reduced the risk of unplanned revascularization (RR, 0.37; 95% CI, 0.28–0.51; *P*<0.0001; I^2^=64.7%). The difference in all‐cause mortality with percutaneous coronary intervention was not statistically significant (RR, 0.85; 95% CI, 0.69–1.04; *P*=0.108; I^2^=0.0%).

**Conclusions:**

For patients with STEMI and multivessel disease, complete revascularization with percutaneous coronary intervention significantly improves hard clinical outcomes including cardiovascular death and myocardial infarction. These data have implications for clinical practice guidelines regarding recommendations for complete revascularization following STEMI.

**Nonstandard Abbreviations and Acronyms**
CAD: coronary artery disease
FFR: fractional flow reserve
PCI: percutaneous coronary intervention
PPCI: primary percutaneous coronary intervention
STEMI: ST‐segment–elevation myocardial infarction

**Clinical Perspective**

**What Is New?**

- Primary percutaneous coronary intervention for patients with ST‐segment–elevation myocardial infarction reduces mortality and myocardial infarction.
- For patients with multivessel coronary artery disease, the optimal treatment of the non‐infarct‐related artery has been controversial.
- For patients with ST‐segment–elevation myocardial infarction and multivessel disease, complete revascularization with percutaneous coronary intervention significantly improves hard clinical outcomes including cardiovascular death and myocardial infarction.

**What Are the Clinical Implications?**

- Clinical guidelines may need to be updated in light of these findings.

Primary percutaneous coronary intervention (PCI) of the infarct‐related artery reduces mortality and myocardial infarction (MI) in patients with ST‐segment–elevation MI (STEMI).^1^ STEMI patients commonly have multivessel coronary artery disease (CAD)^1^, ^2^ and the presence of multivessel disease confers a worse prognosis.^3^

The treatment of non‐infarct related arteries in STEMI patients has been controversial, and previously was considered to be a class III indication^4^, ^5^ outside of the setting of cardiogenic shock, largely on the basis of observational studies.^6^ More recently, randomized controlled trials (RCTs) in the field have suggested that complete revascularization with PCI is safe for these patients and may be beneficial. Guidelines now permit PCI to the non‐infarct‐related artery for STEMI patients but are still somewhat conservative.^7^, ^8^

The RCTs in the field to date and meta‐analyses of them have primarily demonstrated reductions in composite end points (typically major adverse cardiac events, which are defined variably across trials).

With the publication of the largest RCT to date in this field (the COMPLETE [Complete versus Culprit‐Only Revascularization Strategies to Treat Multivessel Disease after Early PCI for STEMI] trial^9^) and longer‐term follow‐up available from another trial,^10^ we sought to perform an up‐to‐date meta‐analysis focusing on individual clinical end points to further evaluate the benefit of complete revascularization with PCI for patients with STEMI and multivessel CAD.

## Methods

The data that support the findings of this study are available from the corresponding author on reasonable request.

We carried out a meta‐analysis of RCTs that evaluated complete revascularization with PCI for patients with STEMI and multivessel disease. The analysis was conducted in accordance with the published PRISMA guidance^11^ and was prospectively registered at the PROSPERO (international prospective register of systematic reviews) (CRD42020149243).

### Search Strategy

We performed a systematic search of the Medline, Cochrane Central Register of Controlled Trials, and Embase databases from September 2019 to January 2020 for all studies of complete revascularization in STEMI. Our search strings included (*STEMI* or *ST‐segment myocardial infarction*) AND *multivessel*; and *percutaneous coronary intervention*, respectively. We also hand‐searched the bibliographies of relevant selected studies, reviews, and meta‐analyses to identify further eligible studies. Abstracts were reviewed for suitability and articles accordingly retrieved. Two independent reviewers performed the search and literature screening (Y.A. and A.A.), with disputes resolved by consensus following discussion with a third author (J.H.).

### Inclusion and Exclusion Criteria

We considered all randomized studies of complete revascularization in STEMI. Studies were eligible if they reported clinical outcome data following randomization to complete or culprit‐only revascularization. Observational and unpublished studies were not considered.

### End Points

The primary efficacy end point was cardiovascular death, and the primary safety end point was risk of major bleeding. We considered MI, all‐cause mortality, unplanned revascularization, and contrast‐induced nephropathy as secondary end points. All analyses were at the latest available follow‐up.

### Data Extraction and Analysis

Two authors (Y.A. and A.A.) independently abstracted the data from included trials, verified by a third author (J.H.). Included studies were assessed using the Cochrane Risk of Bias tool.^12^ Tests for publication bias would be performed only in the event of ≥10 trials being included for analysis, and a Funnel plot would be used.^13^

We analyzed efficacy on an intention‐to‐treat basis. The primary outcome measure was the relative risk (RR) of cardiovascular death. Random‐effects meta‐analyses were performed using the restricted maximum likelihood estimator. Additional analyses were performed using fixed effects. All outcomes were assessed as RRs.

As a secondary analysis, we analyzed cardiovascular death, MI, all‐cause mortality, and unplanned revascularization as hazard ratios when the trials reported these data. We extracted the hazard ratios with their associated 95% CIs and *P* values. A random‐effects meta‐analysis was performed of the natural logarithm of the hazard ratios and their associated standard errors using the restricted maximum likelihood estimator. The standard error was calculated by dividing the difference between the natural logarithms of the upper and lower 95% CIs by 2 times the appropriate normal score (1.96). Where the lower 95% CI level approached zero, the standard error was calculated using only the difference between the natural logarithm of the upper 95% CI level and the natural logarithm of the point estimate.

We used the I^2^ statistic to assess heterogeneity.^14^ Low or mild heterogeneity was defined as 0% to 30%; moderate heterogeneity was defined as 31% to 60%; and >60% was defined as substantial heterogeneity. Mean values are expressed as mean±SD unless otherwise stated. Statistical significance was set at *P*<0.05. The statistical programming environment R^15^ with the *metafor* package^16^ was used for all statistical analysis.

### Subgroups

We specified the timing of complete revascularization (immediate or staged) as a subgroup analysis. Interactions between subgroups were assessed with metaregression using a mixed‐effects model.

## Results

**Figure 1:**
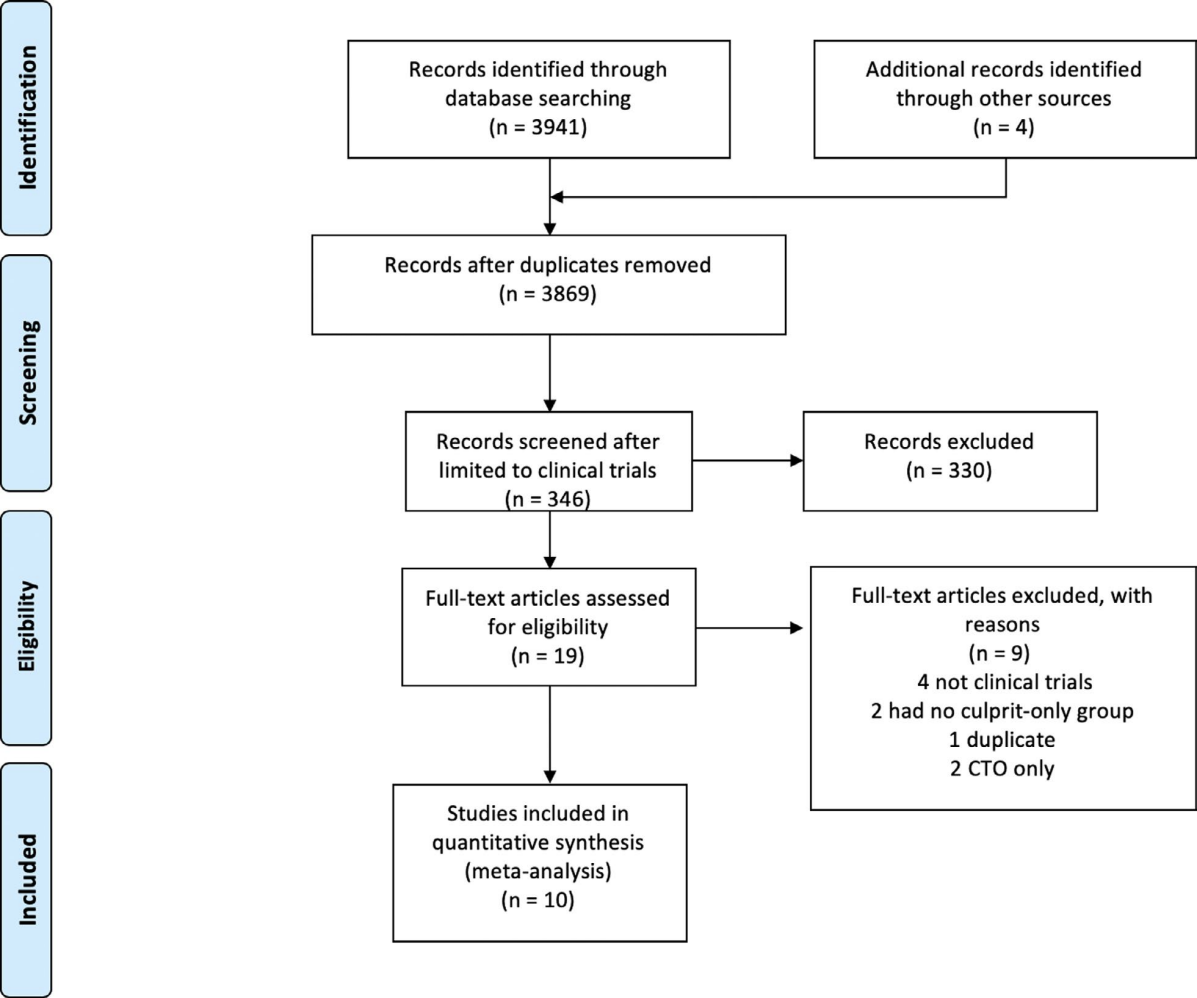
Search strategy and source of included studies. CTO indicates chronic total occlusion.

Ten studies^9^, ^17^, ^18^, ^19^, ^20^, ^21^, ^22^, ^23^, ^24^, ^25^ enrolling 7542 patients met the inclusion criteria (Figure 1). Of those, 3664 patients were randomized to complete revascularization and 3878 to culprit‐only revascularization, with a weighted mean follow‐up of 31.4 months.

Across all studies, the mean age was 62 years. The full characteristics of included studies including follow‐up duration, inclusion criteria, and end points are shown in Table 1, and important differences are highlighted below.

**Table 1:** Characteristics of Included Studies

Author	Study Acronym	Year	Region	N	Mean Age*	Follow‐Up, mo†	Entry Criteria	Complete Revascularization	Culprit‐Only Revascularization	Non‐Culprit‐Vessel Criteria	Primary Efficacy Outcomes	Safety Outcomes
Mehta et al^9^	COMPLETE	2019	31 countries across North America, Europe, Asia and Africa‡	4041	61.6 (±10.7)	35.8 (IQR, 27.6–44.3)	STEMI with culprit primary PCI and at least 1 nonculprit angiographically significant lesion and patient able to be randomized within 72 h of culprit‐lesion PCI	Staged PCI of all nonculprit lesions either during admission or after discharge, ≤45 d from randomization	No further revascularization unless protocol criteria for crossover met	At least 70% stenosis or 50%–69% stenosis with FFR ≤0.80	Composite of cardiovascular death, new MI. Composite of cardiovascular death, new MI, ischemia‐driven revascularization	Major bleeding, contrast‐associated acute kidney injury
Smits et al^17^	Compare‐Acute	2017	24 centers in Europe and Asia	885	62 (±10)	36	STEMI with culprit primary PCI and at least 1 nonculprit artery amenable to PCI	FFR measurement: if ≤0.80, nonculprit revascularization during index admission preferably within 72 h	FFR measurement without revascularization but planned revascularization within 45 d could occur (without knowledge of FFR)	>50% stenosis in major artery or branch vessel >2 mm diameter, FFR ≤0.80	Composite of all‐cause mortality, nonfatal MI, any revascularization, cerebrovascular events	Net adverse clinical events, death from any cause or MI, any bleeding, hospitalization for heart failure, unstable angina r chest pain, revascularization, stent thrombosis
Hamza et a^18^	NA	2016	Not stated (authors’ centers are Egypt and USA)	100	56.4 (±11.5)	6	STEMI in patients with diabetes mellitus undergoing primary PCI with nonculprit stenosis	PCI to all nonangiographically culprit lesions either at time of primary PCI or within 72 h	Not specifically stated	80% stenosis of vessel	Composite of all‐cause mortality, recurrent MI, ischemia‐driven revascularization	Major bleeding, contrast‐induced nephropathy
Zhang et a^19^	NA	2015	Not stated (authors’ centers are in China)	428	NA	NA	STEMI in patients undergoing primary PCI with nonculprit stenoses	Staged PCI to nonculprit vessels 7–10 d after primary PCI	PCI to nonculprit lesions if evidence of ischemia (symptoms, ECG changes, nuclear study)	75%–90% stenosis	All cause mortality, cardiovascular death, MI	Hospitalizations
Engstrøm et al^20^	DANAMI‐3‐PRIMULTI	2015	Denmark	627	63 (34–92)	27 (12–24)	STEMI in patients undergoing primary PCI with >50% stenosis in nonculprit artery	Staged PCI to nonculprit artery if FFR ≤0.80, 2 d after initial PCI	No further revascularization planned	>50% stenosis in vessel >2 mm diameter, FFR ≤0.80	Composite of all‐cause mortality, reinfarction, or ischemia‐driven (subjective or objective) revascularization	Periprocedural MI, bleeding requiring transfusion or surgery, contrast‐induced nephropathy, stroke
Gerschlick et al^10^, ^21^	CvLPRIT	2015	UK	296	64.6 (±11.2)	66 (0–87)	STEMI in patients undergoing primary PCI with nonculprit artery with angiographically significant stenosis	PCI to nonculprit artery during primary PCI procedure	No further revascularization planned	>70% diameter stenosis in 1 plane or >50% in 2 planes in major/branch vessel >2 mm diameter	Composite of all‐cause mortality, recurrent MI, heart failure, revascularization	Cardiovascular death, stroke, major bleeding, contrast‐induced nephropathy
Wald et al^22^	PRAMI	2013	UK	465	62 (32–92)	23	STEMI in patients undergoing primary PCI with nonculprit artery with angiographically significant stenosis	PCI to nonculprit artery during primary PCI procedure	PCI to residual stenoses only if refractory angina and objective ischemia test positive	>50% stenosis in nonculprit artery	Composite of cardiovascular death, nonfatal MI, refractory angina	Noncardiovascular death, repeated revascularisation were secondary outcomes
Dambrink et al^23^	n/a	2010§	Netherlands	121	62 (±10)	36	STEMI in patients undergoing primary PCI with at least 2 angiographically significant stenoses in different vessels (or branch plus vessel)	PCI to nonculprit artery before discharge if FFR positive	Ischemia‐guided additional revascularization if symptomatic (exercise testing, dobutamine stress echocardiography, or myocardial scintigraphy)	>50% stenosis in >2.5 mm vessel if FFR ≤0.75	Ejection fraction	MACE
Politi et al^24^	NA	2010	All authors’ centers are in Italy	263	65.2±12.2	30 (±17)	STEMI in patients undergoing primary PCI with at least 2 angiographically significant stenoses in different vessels	Two arms: (1) staged PCI to nonculprit artery, (2) PCI to nonculprit artery during primary PCI procedure	No further revascularization planned	>70% stenosis	Composite of cardiac or noncardiac death, in‐hospital death, reinfarction, rehospitalization for acute coronary syndrome and repeated coronary revascularization	Contrast‐induced nephropathy
Di Mario et al^25^	HELP AMI	2004	Authors’ centers are in UK and Italy	69	65.3 (±7.4)	12	STEMI with angiographically severe stenosis in at least 2 major vessels	Nonculprit PCI performed during primary PCI procedure	Nonculprit PCI according to physician's discretion based on symptoms and ischemia testing	Major vessel (% not stated) but balloon angioplasty allowed in vessel <2.5 mm if at least 1 main vessel also stented	Repeat revascularization	MACE

Compare Acute indicates Fractional Flow Reserve–Guided Multivessel Angioplasty in Myocardial Infarction; COMPLETE, Complete versus Culprit‐Only Revascularization Strategies to Treat Multivessel Disease after Early PCI for STEMI; CvLPRIT, Complete Versus Lesion‐Only Primary PCI trial; DANAMI 3 PRIMULTI, Complete revascularisation versus treatment of the culprit lesion only in patients with ST‐segment elevation myocardial infarction and multivessel disease; FFR, fractional flow reserve; HELP‐AMI, Hepacoat for Culprit or Multivessel Stenting for Acute Myocardial Infarction; IQR, interquartile range; MACE, major adverse cardiac events; MI, myocardial infarction; NA, not available; PCI, primary catheter intervention; PRAMI, Preventive Angioplasty in Acute Myocardial Infarction; and STEMI, ST‐segment–elevation myocardial infarction. *Mean age, where stated, in years (±SD) or median age (interquartile range) except for PRAMI, where mean (range) is provided; value for complete revascularization group provided where values differ between groups. ^†^Mean follow‐up duration, where stated, in months (±SD where provided) except for COMPLETE and CvLPRIT, where median and IQR are provided, and Compare‐Acute, Hamza et al^18^, and HELP AMI, where follow‐up duration was specified; value for complete revascularization group provided where values differ between groups. ^‡^Majority of patients recruited in Canada and United Kingdom (2293, 56%).

There was some variation in study design between the included trials. The timing of non–culprit vessel PCI in the complete revascularization arms of the trials varied between nonculprit PCI during the primary PCI procedure, staged PCI before discharge from the index admission, staged PCI after discharge, or combinations of these strategies. PRAMI (Preventive Angioplasty in Acute Myocardial Infarction), CvPLRIT (Complete Versus Lesion‐Only Primary PCI) trial and HELP‐AMI (Hepacoat for Culprit or Multivessel Stenting for Acute Myocardial Infarction) all included an arm in which nonculprit PCI was specified to occur during the index primary PCI procedure, whereas COMPLETE allowed staged PCI after discharge up to 45 days after the index procedure. The location, degree, and index vessel diameter thresholds for coronary stenoses to achieve angiographic significance also varied between included studies: PRAMI was the least restrictive, permitting 50% visual stenosis to be an appropriate nonculprit lesion, whereas Hamza et al required 80% stenosis. Compare Acute (Fractional Flow Reserve–Guided Multivessel Angioplasty in Myocardial Infarction), DANAMI‐3‐PRIMULTI (complete revascularisation versus treatment of the culprit lesion only in patients with ST‐segment elevation myocardial infarction and multivessel disease), and Dambrink et al all required fractional flow reserve (FFR) assessment of the stenosis. Definitions of clinical end points used in each trial are shown in Table S1.

Trial quality was assessed using the Cochrane risk‐of‐bias tool and is shown in Table 2. Given the inherent difficulty in sham‐blinding nonculprit PCI, none of the trials adequately blinded the patient or the operator to treatment allocation. However, most outcomes assessed, such as all‐cause mortality, cardiovascular death, and nonfatal MI, are relatively bias‐resistant in this regard, with the exception of unplanned revascularization. There was no evidence of publication bias as assessed by the funnel plot (*P*=0.669; see Figure S1).

**Table 2:** Risk of Bias of Included Studies

Author	Study Acronym	Year	Random Sequence Generation	Allocation Concealment	Blinding of Participants and Personnel	Blinding of Outcome Assessment	Incomplete Outcome Data	Selective Reporting	Other Bias
Mehta et al^9^	COMPLETE	2019	Low risk Computer‐generated system	Low risk Computer‐generated system	Unclear Not specified	Low risk Events adjudicated by independent committee	Low risk Low drop‐out rate	Low risk Pre‐specified outcomes reported	Low risk Partly industry‐funded but these parties not involved in study design or management
Smits et al^17^	Compare‐Acute	2017	Low risk Opaque envelope system	Low risk Opaque envelope system	Unclear Not specified	Low risk Events adjudicated by independent committee	Low risk Low dropout rate	Low risk Prespecified outcomes reported	Low risk Partly industry funded but these parties not involved in study design or management
Hamza et al^18^	NA	2016	Unclear Not specified	Unclear Not specified	Unclear Not specified	Unclear Not specified	Low risk Low dropout rate	High risk Not preregistered and protocol not published	Unclear Source of funding not stated
Zhang et al^19^	NA	2015	Unclear Not specified	Unclear Not specified	Unclear Not specified	Unclear Not specified	Unclear Not specified	High risk Not preregistered and protocol not published	Unclear Source of funding not stated
Engstrøm et al^20^	DANAMI‐3‐PRIMULTI	2015	Low risk Centralized web‐based system	Unclear Not specified	High risk Open‐label study	Low risk Outcomes adjudicated by independent events committee	Low risk Low dropout rates	Low risk Prespecified outcomes reported	Low risk Funded by independent body
Gerschlick et al^10^, ^21^	CvLPRIT	2015	Low risk Interactive voice‐response program	Low risk Automated telephone randomisation	High risk Open label	Low risk Outcome adjudication by blinded clinicians	High risk Low dropout rates in both groups but low event rate	Low risk Prespecified outcomes reported	Low risk Funded by independent body
Wald et al^22^	PRAMI	2013	Low risk Computer generated	Unclear Not specified	High risk Open label for participants	Low risk Blinded adjudication	High risk Low dropout rates in both groups but low event rate	Low risk Prespecified outcomes reported	High risk Early termination (significant between groups difference in primary outcome)
Dambrink et al^23^	n/a	2010	Low risk Computer‐based randomization	Unclear Not specified	Unclear Not specified	Unclear Not specified for primary outcomes	Low risk Low rates of dropout	High risk Not preregistered and protocol not published	High risk Early termination (due to slow enrollment), source of funding not stated
Politi et al^24^	n/a	2010	Low risk Computerized randomization	Unclear Not specified	Unclear Not specified	Unclear Not specified	Unclear Not specified	High risk Not preregistered and protocol not published	Unclear Source of funding not stated
Di Mario et al^25^	HELP AMI	2009	Unclear Not specified	Unclear Not specified	Unclear Not specified	Unclear Not specified	Unclear Not specified	High risk Not preregistered and protocol not published	Unclear Source of funding not stated

A summary of stent types used in the included trials is shown in Data S1.

### Efficacy of Complete Versus Culprit‐Only Revascularization

#### Cardiovascular Death

**Figure 2:**
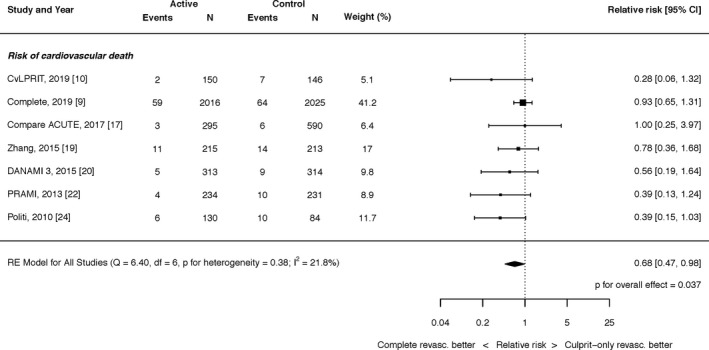
Effect of complete revascularization on cardiovascular death. Compare Acute indicates Fractional Flow Reserve–Guided Multivessel Angioplasty in Myocardial Infarction; COMPLETE, Complete versus Culprit‐Only Revascularization Strategies to Treat Multivessel Disease after Early PCI for STEMI; CvLPRIT, Complete Versus Lesion‐Only Primary PCI trial; DANAMI 3 PRIMULTI, Complete revascularisation versus treatment of the culprit lesion only in patients with ST‐segment–elevation myocardial infarction and multivessel disease; PRAMI, Preventive Angioplasty in Acute Myocardial Infarction.

Complete revascularization with PCI resulted in a significant reduction in the risk of cardiovascular death (RR, 0.68; 95% CI, 0.47–0.98; *P*=0.037; Figure 2). There was low heterogeneity (I^2^=21.8%).

#### Myocardial Infarction

**Figure 3:**
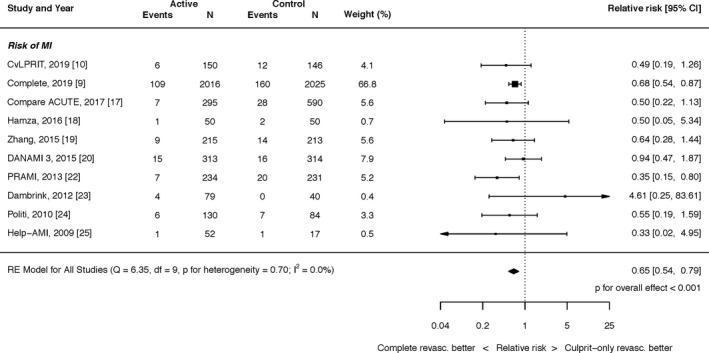
Effect of complete revascularization on myocardial infarction. Compare Acute, Fractional Flow Reserve–Guided Multivessel Angioplasty in Myocardial Infarction; COMPLETE, Complete versus Culprit‐Only Revascularization Strategies to Treat Multivessel Disease after Early PCI for STEMI; CvLPRIT, Complete Versus Lesion‐Only Primary PCI trial; DANAMI 3 PRIMULTI, Complete revascularisation versus treatment of the culprit lesion only in patients with ST‐segment–elevation myocardial infarction and multivessel disease; HELP‐AMI, Hepacoat for Culprit or Multivessel Stenting for Acute Myocardial Infarction; PRAMI, Preventive Angioplasty in Acute Myocardial Infarction.

Complete revascularization with PCI resulted in a significant reduction in the risk of MI (RR, 0.65; 95% CI, 0.54–0.79; *P*<0.0001; Figure 3). There was no heterogeneity (I^2^=0.0%). This result was unchanged by restricting the inclusion to patients with spontaneous MI (RR, 0.58; 95% CI, 0.46–0.73; *P*<0.001; I^2^=0.0%; Figure S2).

#### All‐Cause Mortality

**Figure 4:**
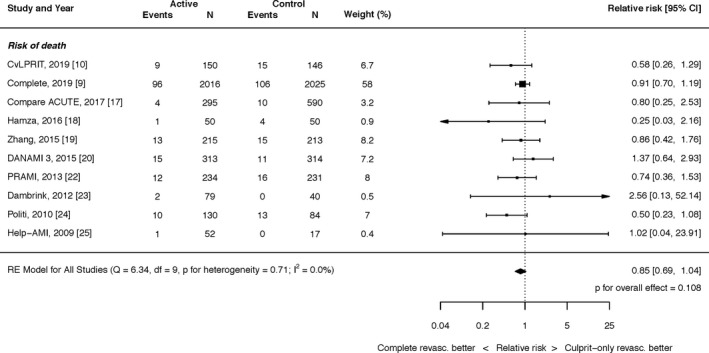
Effect of complete revascularization on all‐cause mortality. Compare Acute, Fractional Flow Reserve–Guided Multivessel Angioplasty in Myocardial Infarction; COMPLETE, Complete versus Culprit‐Only Revascularization Strategies to Treat Multivessel Disease after Early PCI for STEMI; CvLPRIT, Complete Versus Lesion‐Only Primary PCI trial; DANAMI 3 PRIMULTI, Complete revascularisation versus treatment of the culprit lesion only in patients with ST‐segment–elevation myocardial infarction and multivessel disease; HELP‐AMI, Hepacoat for Culprit or Multivessel Stenting for Acute Myocardial Infarction; PRAMI, Preventive Angioplasty in Acute Myocardial Infarction.

The effect of complete revascularization with PCI on all‐cause mortality was an RR of 0.85 (95% CI, 0.69–1.04; *P*=0.108; Figure 4). There was no heterogeneity (I^2^=0.0%).

#### Unplanned Revascularization

**Figure 5:**
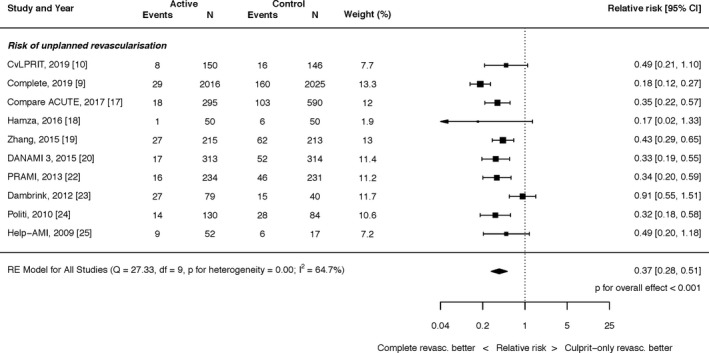
Effect of complete revascularization on unplanned revascularization. Compare Acute, Fractional Flow Reserve–Guided Multivessel Angioplasty in Myocardial Infarction; COMPLETE, Complete versus Culprit‐Only Revascularization Strategies to Treat Multivessel Disease after Early PCI for STEMI; CvLPRIT, Complete Versus Lesion‐Only Primary PCI trial; DANAMI 3 PRIMULTI, Complete revascularisation versus treatment of the culprit lesion only in patients with ST‐segment–elevation myocardial infarction and multivessel disease; HELP‐AMI, Hepacoat for Culprit or Multivessel Stenting for Acute Myocardial Infarction; PRAMI, Preventive Angioplasty in Acute Myocardial Infarction.

Complete revascularization with PCI resulted in a significant reduction in the risk of unplanned revascularization (RR, 0.37; 95% CI, 0.28–0.51; *P*<0.0001; Figure 5). There was significant heterogeneity (I^2^=64.7%).

#### Safety of Complete Revascularization

**Figure 6:**
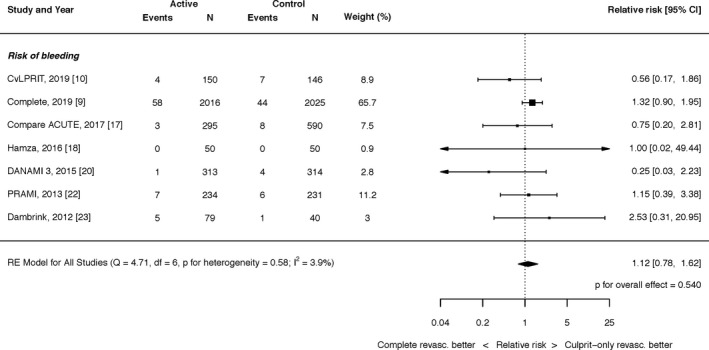
Effect of complete revascularization on major bleeding. Compare Acute, Fractional Flow Reserve–Guided Multivessel Angioplasty in Myocardial Infarction; COMPLETE, Complete versus Culprit‐Only Revascularization Strategies to Treat Multivessel Disease after Early PCI for STEMI; CvLPRIT, Complete Versus Lesion‐Only Primary PCI trial; DANAMI 3 PRIMULTI, Complete revascularisation versus treatment of the culprit lesion only in patients with ST‐segment–elevation myocardial infarction and multivessel disease; PRAMI, Preventive Angioplasty in Acute Myocardial Infarction.

The effect of complete revascularization with PCI on major bleeding was an RR of 1.12 (95% CI, 0.78–1.62; *P*=0.540; Figure 6). There was minimal heterogeneity (I^2^=3.9%). The effect of complete revascularization with PCI on contrast‐induced nephropathy was an RR of 1.42 (95% CI, 0.88–2.30; *P*=0.152; I^2^=0.0%; Figure S3).

#### Impact of Timing of Complete Revascularization

Six trials^16^, ^17^, ^20^, ^21^, ^23^, ^24^ reported outcomes for all‐cause mortality, MI, and unplanned revascularization in patients who underwent immediate complete revascularization. Four trials^16^, ^20^, ^21^, ^23^ reported outcomes for cardiovascular death in patients who underwent immediate revascularization. Five trials^9^, ^18^, ^19^, ^22^, ^23^ reported outcomes for all‐cause mortality, MI, and unplanned revascularization in patients who underwent staged complete revascularization. Four trials^9^, ^18^, ^19^, ^23^ reported outcomes for cardiovascular death in patients who underwent staged revascularization. Staged complete revascularization was performed within a wide temporal interval, from during the index admission up to 45 days after the initial PCI procedure.

**Figure 7:**
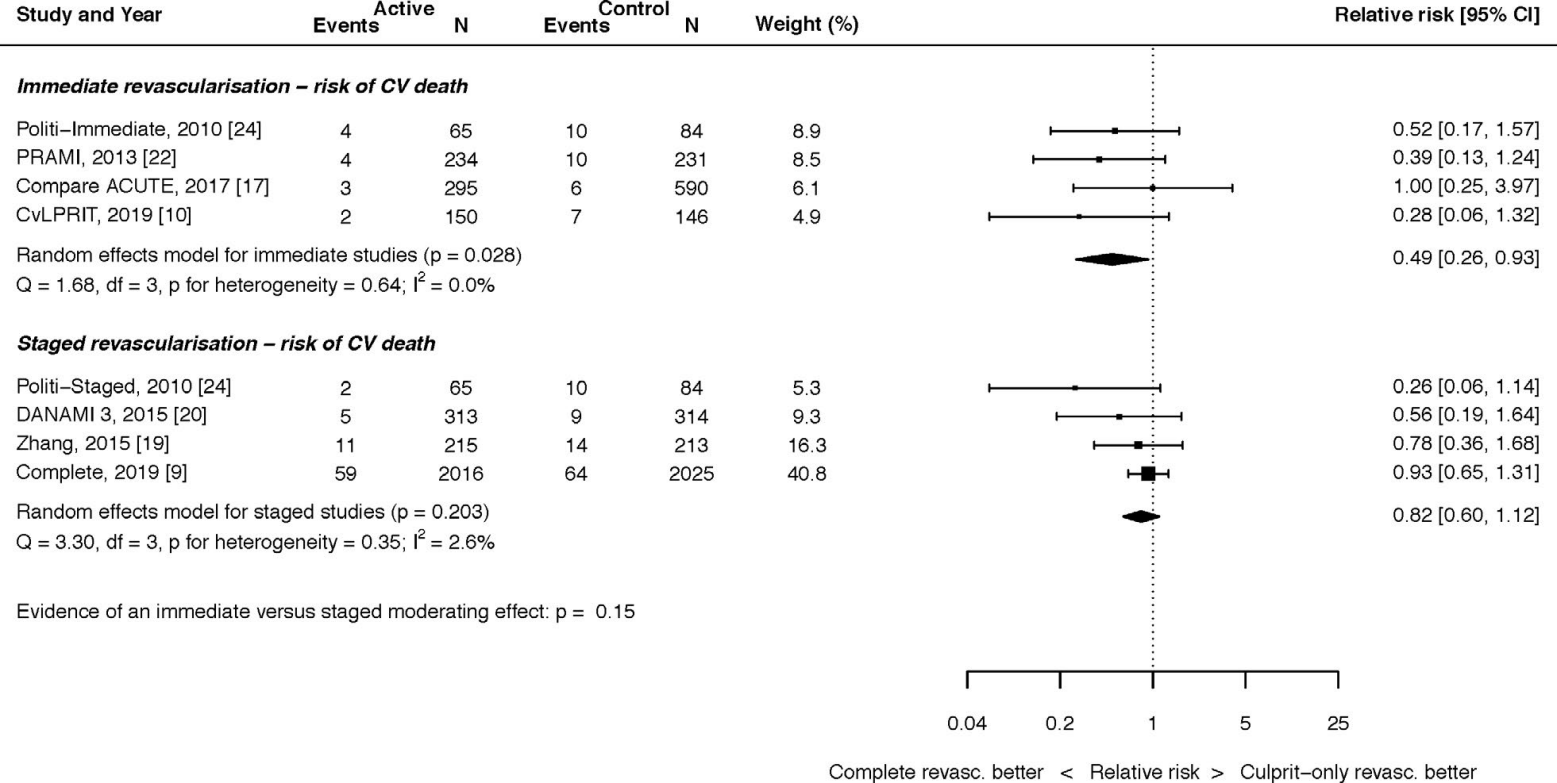
Effect of timing of complete revascularization on cardiovascular (CV) death. Compare Acute, Fractional Flow Reserve–Guided Multivessel Angioplasty in Myocardial Infarction; COMPLETE, Complete versus Culprit‐Only Revascularization Strategies to Treat Multivessel Disease after Early PCI for STEMI; CvLPRIT, Complete Versus Lesion‐Only Primary PCI trial; DANAMI 3 PRIMULTI, Complete revascularisation versus treatment of the culprit lesion only in patients with ST‐segment–elevation myocardial infarction and multivessel disease; PRAMI, Preventive Angioplasty in Acute Myocardial Infarction.

Subgroup analysis did not demonstrate evidence of a significant interaction between the timing of complete revascularization and reduction in cardiovascular death (*P*=0.15; Figure 7).

Subgroup analysis did not demonstrate evidence of a significant interaction between the timing of complete revascularization and the reduction of unplanned revascularization (*P*=0.86). Subgroup analysis also did not demonstrate evidence of a significant interaction between the timing of complete revascularization and the reduction of MI, but the *P* value was borderline (0.05). These plots are shown in Figures S4 and S5.

### Impact of Revascularization Guided by FFR

Three trials^16^, ^19^, ^22^ reported outcomes for all‐cause mortality, MI, and unplanned revascularization in patients who underwent complete revascularization guided by FFR. Two trials^16^, ^19^ reported outcomes for cardiovascular death in patients who underwent complete revascularization guided by FFR. Seven trials^9^, ^17^, ^18^, ^20^, ^21^, ^23^, ^24^ reported outcomes for all‐cause mortality, MI, and unplanned revascularization in patients who underwent complete revascularization guided by angiography. Five trials^9^, ^18^, ^20^, ^21^, ^23^ reported outcomes for cardiovascular death in patients who underwent complete revascularization guided by angiography. The COMPLETE trial was regarded as using an angiographic‐guided approach because only a very small proportion (0.8%) of patients had treatment guided by FFR.

Subgroup analysis did not demonstrate evidence of a significant interaction between the FFR versus angiography‐guided revascularization for any of the end points. Forest plots for each of these end points are shown in Figures S6 through S9.

### Hazard Ratio Analysis

We performed a secondary analysis looking at the efficacy end points using hazard ratios, which is more appropriate for time‐to‐event data but is limited by the reporting of the individual trials. Five trials reported hazard ratios for cardiovascular death, all‐cause mortality, MI, and unplanned revascularization. The results are consistent with the main RR analysis for the end points of MI and unplanned revascularization, and the effect sizes were very similar for cardiovascular death, although they failed to reach statistical significance in light of the smaller sample size. These plots are shown in Figures S10 through S13.

### Fixed‐Effects Analyses

We performed an additional analysis looking at fixed‐effects analyses for all our main end points, the results of which are consistent with our random‐effects analyses, and the plots are shown in Figures S14 through S18.

### Sensitivity Analyses

We performed a sensitivity analysis including only trials assessed as being at low risk of bias. The results are consistent with the main analysis. These plots (for cardiovascular death, MI, all‐cause mortality, and unplanned revascularization) are available in Figures S19 through S22.

We also performed sensitivity analyses excluding trials with low use of drug‐eluting stents (defined as <50% of the total trial population). These results are shown in Figures S23 through S27 and are consistent with the main analysis.

We performed a further *jackknife* or *leave one out* sensitivity analysis, excluding each individual included trial in turn. These plots (for cardiovascular death, MI, all‐cause mortality, and unplanned revascularization) are available in Figures S28 through S64.

## Discussion

In this study we have shown (1) that for patients with STEMI and multivessel disease, the risk of cardiovascular death is reduced by complete revascularization (RR, 0.68; 95% CI, 0.47–0.98; *P*=0.037), and (2) that this reduction in cardiovascular death is may partially be driven by a reduction in MI, which has a similar pooled point estimate (RR, 0.65; 95% CI, 0.54–0.79; *P*<0.0001).

### Superiority of Complete Revascularization to Culprit‐Only Revascularization

The individual trials included in this meta‐analysis have shown reduction in unplanned revascularization with a strategy of complete revascularization after STEMI. This finding is intuitive because all patients in the culprit‐only arm, by eligibility criteria, had angiographically severe stenoses amenable to PCI, and cardiologists were not blinded to their allocation to the culprit‐only arms. Some trials also demonstrated a reduction in MI, including the most recent COMPLETE trial,^9^ which is the largest trial in the field to date. In the current era of contemporary pharmacotherapy and continued advances in stent technology and implantation techniques, hard event rates are low. This makes it difficult for any individual trial in the field of STEMI to show benefits in terms of mortality end points. Consequently, we must turn to meta‐analysis to synthesize all available trial data.

By doing so, we are now able to observe, for the first time, a statistically significant benefit to complete revascularization in STEMI for the end point of cardiovascular death. The mechanism of this reduction in cardiovascular death might be driven by a reduction in MI, particularly as the effect size is similar for these 2 end points. Other possible mechanisms include reduction in ischemia‐driven arrhythmias and heart failure, but no definitive causation can be determined from this analysis.

Our analysis did not demonstrate a statistically significant benefit for complete revascularization with PCI in terms of all‐cause mortality (RR, 0.84; 95% CI, 0.69–1.04; *P*=0.113). This may be due to insufficient power, and future trials in the field may help to identify a benefit in terms of all‐cause mortality, which is the most bias‐resistant end point. There was no heterogeneity for this outcome, and in fact heterogeneity was also low or absent for MI and cardiac death. This implies consistent findings across the included studies and strengthens the conclusions of our analysis.

### Implications for Clinical Practice

It is important that the results of these trials, and the current analysis, are not conflated with the treatment of stable angina, for which PCI should still generally be offered with the goal of alleviating symptoms.^25^ Moreover, this analysis serves to further illustrate the marked differences between patients who have had STEMI and those who have stable angina or stable CAD. The 2 entities are pathophysiologically and biologically distinct and therefore require distinct therapeutic strategies.

Clinicians treating patients with STEMI and multivessel disease have, broadly, 3 different management strategies to choose from: stenting the infarcted artery only and leaving all residual disease to medical therapy (culprit‐only PCI), treating all appropriate stenoses at the time of STEMI (immediate complete revascularization), and treating the infarct‐related artery at the time of STEMI and tackling the residual disease during another procedure (staged complete revascularization).

We sought to investigate whether the timing of complete revascularization had an impact on clinical outcomes. Subgroup analyses did not demonstrate evidence of a significant interaction between the timing of intervention in our analysis; that is, there was a consistent treatment effect for complete revascularization versus infarct‐related artery PCI, regardless of the timing when complete revascularization was achieved. Furthermore, the largest RCT in the field to date (COMPLETE) had no immediate PCI arm (patients underwent PCI to achieve complete revascularization in a staged procedure, either during the hospital admission or as an outpatient within 45 days). A further analysis from the COMPLETE trial, initially presented at Transcatheter Therapeutics 2019 and published subsequently,^26^ did not demonstrate a difference between complete revascularization during the index admission (median, 1 day), or after discharge from the hospital (median, 23 days), with a *P* value for interaction of 0.62 for the outcome of cardiac death or new MI.

It is unlikely that a group in that trial undergoing immediate complete revascularization with PCI would have had better outcomes than a group undergoing staged PCI a median of 1 day after the index procedure. We suggest that achieving complete revascularization, rather than timing of it, is the most important determination of clinical outcomes for these patients. This is also supported by the fact we did not observe a significant interaction whether complete revascularization was guided by FFR or angiography.

Our analysis has not suggested any safety concerns regarding complete revascularization. There was no significant increase in major bleeding or acute kidney injury. These data are reassuring, but treating clinicians must weigh the benefits of complete revascularization (reduction in cardiac death, myocardial infarction, and future revascularization) against potential risks (both short and long term) on an individual case‐by‐case basis. Our analysis demonstrates a reduction in MI with complete revascularization. The ISCHEMIA trial presentation has suggested that in stable CAD, invasive therapy leads to greater procedural MI but less spontaneous MI. This cannot necessarily be extrapolated to the patient population studied in this analysis, but future trials may wish to separately report periprocedural and spontaneous MI in all patients to permit a more nuanced interpretation of the results and to better advise patients on potential risks and benefits.

### Implications for Clinical Practice Guidelines

PCI of the non‐infarct‐related artery was previously given a class III recommendation in guideline documents, but as further RCTs emerged, guideline recommendations were updated.

European guidelines from 2017^7^ now give a IIa recommendation (level of evidence, A) and state that “routine revascularization of non‐infarct‐related artery lesions should be considered in STEMI patients with multivessel disease before hospital discharge.” American College of Cardiology and American Heart Association guidelines from 2015^5^ give a IIb recommendation (level of evidence, B‐R) and state that “PCI of a non‐infarct artery may be considered in selected patients with STEMI and multivessel disease who are hemodynamically stable, either at the time of primary PCI or as a planned procedure.”

On the basis of the totality of the randomized trial data and this analysis, guidelines should be updated to give a class I recommendation for complete revascularization in appropriate STEMI patients.

### Prior Work in the Field

Our meta‐analysis differs from previous analyses in several ways. First, and most obviously, it includes the COMPLETE trial, which is by some margin the largest study in the field; we have also included long‐term follow‐up from the CvLPRIT trial. Second, we used individual end points rather any composite measures such as major adverse cardiac events. The use of composite measures for such an analysis is problematic. If the hazard ratios are synthesized for major adverse cardiac events or the primary composite end point, as it is defined in each individual trial, this will be hampered by the varying definitions seen in each trial. Essentially, disparate data will be meta‐analyzed. If events from individual clinical end points counting and combined to assess major adverse cardiac events or another composite, then there is a risk of counting events twice when the trial is providing time‐to‐event data. Third, we included an analysis of hazard ratios where these data were available, which is the most appropriate analysis for time‐to‐event data.^27^

### Limitations

We could only report the available data. Subgroup analyses based on factors such as location of MI, diabetes mellitus, left ventricular function, location, and complexity of residual CAD was not possible because trials did not uniformly report these data, and if they did, it was only for the primary outcome measure, which differed across each trial. The individual trials also had other differences in methodology and reporting, but this problem is common to all meta‐analyses. It would benefit clinical trialists to attempt to harmonize their definitions of events and their outcome measures to facilitate more accurate synthesis of their results.

The majority of trials did not routinely report postprocedure elevations in cardiac enzymes, so it was not possible to analyze them. The DANAMI trial reported 2 periprocedural MIs in the complete revascularization group but without any details on enzyme elevations; the trial by Dambrink et al reported 4 periprocedural MIs in the complete revascularization group.

Sicker, higher risk patients were generally excluded from these trials. Consequently, our results cannot be extrapolated to patients with cardiogenic shock or those with left main CAD or chronic total occlusions.

Time‐to‐event data are best analyzed using hazard ratios or survival plots. When we performed this analysis, the benefit of complete revascularization remained for MI and revascularization but was not statistically significant for cardiac death. This is likely due to the reduced sample size because not all trials provided hazard ratios or survival plots. If hazard ratios were available for all included studies, the primary end point may have reached statistical significance using hazard ratios, but these data were not available.

## Conclusions

For patients with STEMI and multivessel disease, complete revascularization with PCI significantly improves hard clinical outcomes including cardiovascular death and MI. These data have implications for clinical practice guidelines regarding recommendations for complete revascularization following STEMI.

## Sources of Funding

None.

## Disclosures

Dr Cook and Dr Al‐Lamee have conducted teaching sessions supported by Volcano Corp. Dr Sen has attended and conducted teaching sessions supported by Volcano Corp, St Jude Medical, Medtronic, Pfizer, and AstraZeneca; has received research grant support from Philips, AstraZeneca, Medtronic, and Pfizer; and has received speaking honoraria from Pfizer and Volcano‐Philips. Manish A. Parikh: Speakers bureau—Medtronic, Boston Scientific, Abbott Vascular, CSI; advisory board—Philips, Abbott Vascular, Medtronic. A. Kirtane: Institutional funding to Columbia University and/or Cardiovascular Research Foundation from Medtronic, Boston Scientific, Abbott Vascular, Abiomed, CSI, CathWorks, Siemens, Philips, ReCor Medical; personal: conference honoraria and travel/meal reimbursements only. Ziad A. Ali: institutional research grants to Columbia University from St. Jude Medical, and Cardiovascular Systems Inc. Consultant to St Jude Medical, ACIST. Dimitri Karmpaliotis: Speaker's bureau ‐ Abbott Vascular, Boston Scientific, Medtronic; consultant ‐ Vascular Solutions. The remaining authors have no disclosures to report.

## Supporting information


**Data S1 Table S1 Figures S1–S64 References 9, 16–24**
