# Supplementary material for: Complete Revascularization by Percutaneous Coronary Intervention for Patients With ST‐Segment–Elevation Myocardial Infarction and Multivessel Coronary Artery Disease: An Updated Meta‐Analysis of Randomized Trials
Source: J Am Heart Assoc. 2020 Jun 1;9(12):e015263. doi: 10.1161/JAHA.119.015263 (PMC7429036; doi:10.1161/JAHA.119.015263)

SUPPLEMENTAL MATERIAL

Data S1.

Summary of use of stent types in included trials:

In the COMPLETE trial, 86.4% of patients in the complete revascularization arm and 86.1% of the culprit-only arm received a drug-eluting stent during the index procedure. A breakdown of different drug-eluting stent types was not provided.

In the COMPARE-ACUTE trial, 95.4% in the complete revascuarization arm received a DES, and in the culprit-only arm 96.1% received DES. In the complete arm, this was broken down to 227 Xience (72.8%), 6 Promus (1.9%), 79 Other DES (25.3%); and in the culprit arm this was broken down to 442 Xience (71.3%), 20 Promus (3.2%), 158 Other DES (25.5%).

In the CvLPRIT trial, 95.9% of patients in the complete revascularization arm and 90.7% of the culprit-only arm received a drug-eluting stent. A breakdown of different drug-eluting stent types was not provided.

In the DAMBRINK trial, 22.5% in the complete revascularization arm and 17.1% in the culprit-only arm received a drug-eluting stent.

In the DANAMI trial, 93% in the complete revascularization arm and 95% in culprit-only arm received a drug-eluting stent.

In the Hamza trial, drug-eluting stents were used in all patients.

In the HELP-AMI trial, the heparin-coated Bx velocity stents were used in all patients.

In the Politi trial, 16.9% in the complete revascularization arm and 11.9% in culprit-only arm received a drug-eluting stent.

In the PRAMI trial, 63% in the complete revascularization arm and 58% in the culprit-only arm received a drug-eluting stent.

Table S1. Endpoint definitions.

Author	Study Acronym	Definition of CV Death	Definition of MI	Definition of IDR
Mehta *et al*[9]	COMPLETE	Clear CV or unknown cause of death. Documented non-CV deaths classified as non-CV (e.g. cancer)	Abnormal troponin + one of new symptoms, new ST-T change / LBBB / Q waves, new RWMA / non-viable myocardium on imaging or autopsy/angiographic intra-coronary/stent thrombus. Cardiac death with symptoms and ST-T change / LBBB but death prior to troponin measurement. Peri-PCI MI: troponin >35x ULN / CK-MB >5x ULN + one of new symptoms, new ST-T change / LBBB, new RWMA / non-viable myocardium on imaging or evidence of PCI complication. Peri-CABG: troponin >70x ULN / CK-MB >10x ULN + one of new q waves / LBBB, new graft/native vessel occlusion or new RWMA / non-viable myocardium on imaging.	All of the following: 1) CCS class ≥2 angina despite GDMT, 2) PCI / CABG of culprit lesion (within 5mm of stented segment) or non-culprit elsion that resulted in trial eligibility, 3) one of: positive functional study demonstrating reversible ischaemia, new ischaemic ECG changes consistent with a coronary territory or FFR ≤ 0.8.
Smits *et al.*[16]	Compare-Acute	CV death not reported	Rise and fall of troponin / CK-MB + one of symptoms, q waves, ST elevation / depression. Q waves without CK-MB rise. Confirmed MI without Q waves.<br><br>Peri-PCI MI: rise of CK-MB >3x ULN within 48 hours.<br>Peri-CABG PCI: rise of CK-MB >5x ULN within 7 days.<br>If peak CK/CK-MB from index infarct not reached: chest pain >20 minutes, or new ecg changes, with peak CK/CK-MB 24 hours later ≥50% higher. If CK/CK-MB falling or normalised within 24 hours of index PCI: new rise >2x ULN if normalised or >50% nadir if falling.	Any revascularisation (not IDR)
Hamza *et a*[17]	n/a	CV death not reported.	Not stated	Not stated
Zhang *et al*[18]	n/a	Not translated	Not translated	Revascularisation not reported.
Engstrøm *et al.*[19]	DANAMI-3-PRIMULTI	Not stated	Not stated	ischaemia-driven (subjective or objective) revascularisation of lesions in non-infarct related arteries

Gerschlick *et al.*[20]	CvLPRIT	Any cardiac causes, or other vascular causes (e.g. pulmonary embolism, aortic dissection)	Type 1: Spontaneous re-MI: Recurrent angina symptoms or new ECG changes occurring before PCI or <48 hours from PCI compatible with re-MI with an elevation of CK-MB, troponin, or total CK above ULN and 20% higher than previous value.<br>Type 4a: CK-MB or total CK >3 times the ULN within 48 hours following PCI. If the pre-PCI CK-MB or total CK level > ULN, also: either falling CK-MB or total CK level prior to the onset of the suspected event, or a peak of biomarker ≥ 20% above the previous value. With appropriate clinical presentation or new ischemic ECG changes (ST elevation/depression or new Q waves/LBBB).<br>Type 4b: MI associated with stent thrombosis on angiography/autopsy as well as fulfilling the criteria of spontaneous MI (Type 1)	Target lesion re-interventions: inside or within 5 mm of stent. Target vessel revascularisation: repeated interventions in the same vessel by PCI/CABG. PCI to lesions not identified previously. CABG for new symptoms or complications of PCI.
Wald *et al.*[21]	PRAMI	Not stated	Symptoms of cardiac ischemia and a troponin > ULN. For patients with a recurrent MI within 14 days after randomization, the definition required new ST change or LBBB with angiographic evidence of coronary-<br>artery occlusion	Repeat revascularisation was a secondary outcome (not IDR).
Dambrink *et al.*[22]	n/a	Not reported	New Q-waves or a new CK and CK-MB rise > ULN (including peri-procedural MI)	Additional unplanned revascularisations reported (not IDR)
Politi *et al.*[23]	n/a	Not stated	Not stated	Not stated
Di Mario *et al.*[24]	HELP AMI	Not stated	Not Stated	Not Stated

CV – cardiovascular, MI – myocardial infarction, RWMA – regional wall motion abnormality, LBBB – left bundle branch block, IDR – ischaemia driven revascularisation, PCI – percutaneous catheter intervention, CABG – Coronary Artery Bypass Grafting, CCS – Canadian Cardiovascular Society, GDMT – guideline directed medical therapy

Figure S1. Funnel plot for publication bias.

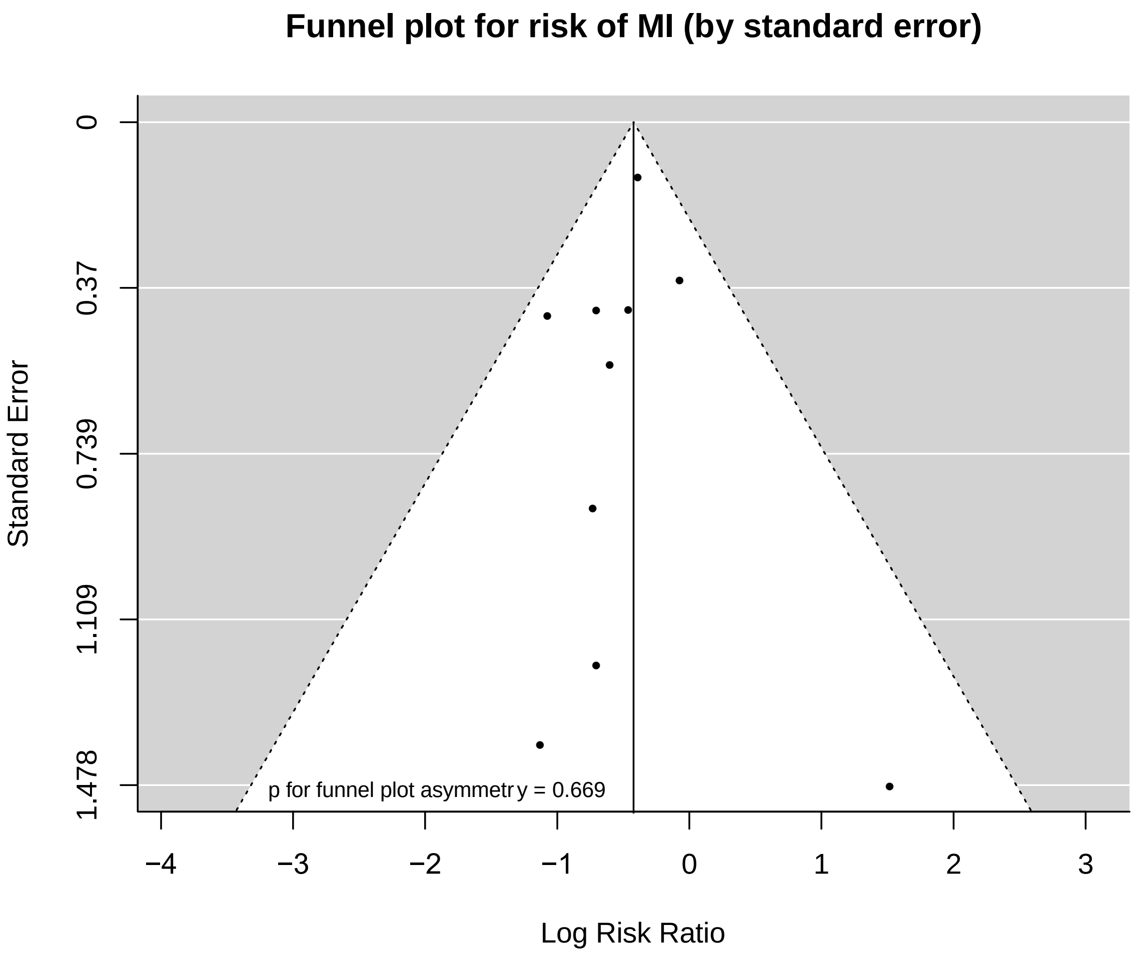

Figure S2. Effect of complete revascularization on risk of spontaneous myocardial infarction.

Study and Year	Active Events	Active N	Control Events	Control N	Weight (%)	Relative risk [95% CI]
Risk of spontaneous MI						
CvLPRIT, 2019	0	150	2	146	0.6	0.19 [0.01, 4.02]
Complete, 2019	83	2016	142	2025	75.3	0.59 [0.45, 0.76]
Compare ACUTE, 2017	5	295	17	590	5.4	0.59 [0.22, 1.58]
DANAMI 3, 2015	13	313	16	314	10.3	0.82 [0.40, 1.67]
PRAMI, 2013	7	234	20	231	7.4	0.35 [0.15, 0.80]
Dambrink, 2012	0	79	0	40	0.3	0.51 [0.01, 25.36]
Help−AMI, 2009	1	52	1	17	0.7	0.33 [0.02, 4.95]
RE Model for All Studies ($Q = 3.01$, $df = 6$, p for heterogeneity = 0.81; $I^2 = 0.0\%$)						0.58 [0.46, 0.73]

p for overall effect < 0.001

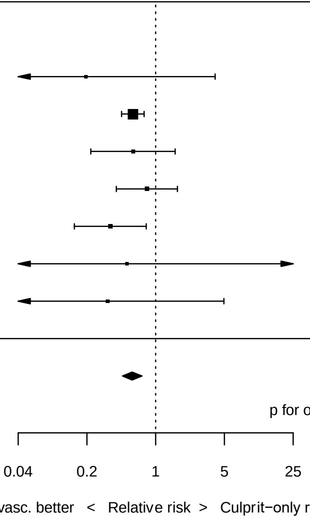

Figure S3. Effect of complete revascularization on risk of contrast-induced nephropathy.

Study and Year	Active Events	Active N	Control Events	Control N	Weight (%)	Relative risk [95% CI]
Risk of contrast induced nephropathy						
CvLPRIT, 2019	2	150	2	146	6.1	0.97 [0.14, 6.82]
Complete, 2019	30	2016	19	2025	70.6	1.59 [0.90, 2.81]
Hamza, 2016	3	50	1	50	4.6	3.00 [0.32, 27.87]
DANAMI 3, 2015	4	313	1	314	4.8	4.01 [0.45, 35.70]
PRAMI, 2013	1	234	3	231	4.5	0.33 [0.03, 3.14]
Politi, 2010	3	130	3	84	9.3	0.65 [0.13, 3.13]
RE Model for All Studies (Q = 4.16, df = 5, p for heterogeneity = 0.53; I^2 = 0.0%)						1.42 [0.88, 2.30]

p for overall effect = 0.152

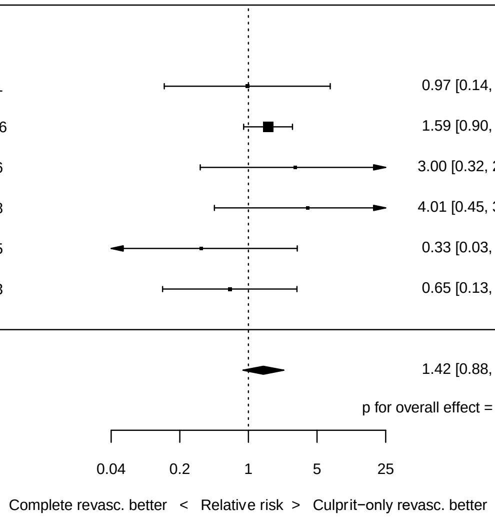

Figure S4. Effect of timing of complete revascularization on myocardial infarction.

Study and Year	Active Events	Active N	Control Events	Control N	Weight (%)	Relative risk [95% CI]
Immediate revascularisation − risk of MI						
Help−AMI, 2009	1	52	1	17	0.5	0.33 [0.02, 4.95]
Politi−Immediate, 2010	2	65	7	84	1.6	0.37 [0.08, 1.72]
PRAMI, 2013	7	234	20	231	5.2	0.35 [0.15, 0.80]
Hamza, 2016	1	50	2	50	0.7	0.50 [0.05, 5.34]
Compare ACUTE, 2017	7	295	28	590	5.5	0.50 [0.22, 1.13]
CvLPRIT, 2019	6	150	12	146	4	0.49 [0.19, 1.26]
Random effects model for immediate studies (p < 0.001)						0.43 [0.27, 0.68]
Q = 0.55, df = 5, p for heterogeneity = 0.99; I^2 = 0.0%						
Staged revascularisation − risk of MI						
Politi−Staged, 2010	4	65	7	84	2.6	0.74 [0.23, 2.42]
Dambrink, 2012	4	79	0	40	0.4	4.61 [0.25, 83.61]
DANAMI 3, 2015	15	313	16	314	7.8	0.94 [0.47, 1.87]
Zhang, 2015	9	215	14	213	5.5	0.64 [0.28, 1.44]
Complete, 2019	109	2016	160	2025	66.2	0.68 [0.54, 0.87]
Random effects model for immediate studies (p = 0.001)						0.71 [0.58, 0.88]
Q = 2.41, df = 4, p for heterogeneity = 0.66; I^2 = 0.0%						

Evidence of an immediate versus staged moderating effect: p = 0.05

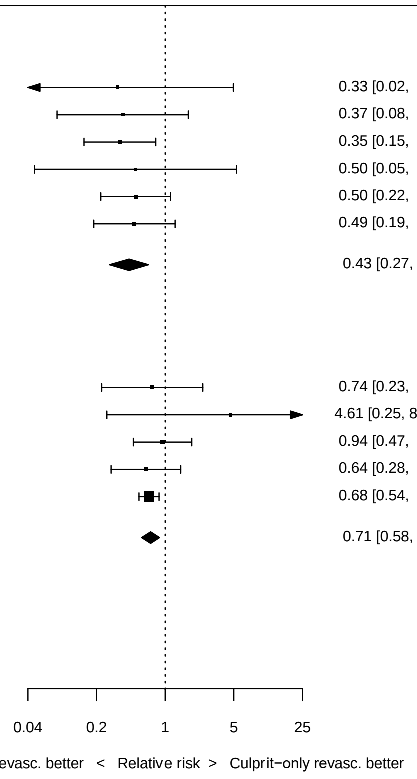

Figure S5. Effect of timing of complete revascularization on unplanned revascularization.

Study and Year	Active Events	Active N	Control Events	Control N	Weight (%)	Relative risk [95% CI]
Immediate revascularisation − risk of unplanned revascularisation						
Help−AMI, 2009	9	52	6	17	6.7	0.49 [0.20, 1.18]
Politi−Immediate, 2010	6	65	28	84	7.2	0.28 [0.12, 0.63]
PRAMI, 2013	16	234	46	231	10.6	0.34 [0.20, 0.59]
Hamza, 2016	1	50	6	50	1.8	0.17 [0.02, 1.33]
Compare ACUTE, 2017	18	295	103	590	11.4	0.35 [0.22, 0.57]
CvLPRIT, 2019	8	150	16	146	7.2	0.49 [0.21, 1.10]
Random effects model for immediate studies ($p < 0.001$)						0.36 [0.27, 0.48]
$Q = 1.96$, df = 5, p for heterogeneity = 0.85; $I^2 = 0.0\%$						
Staged revascularisation − risk of unplanned revascularisation						
Politi−Staged, 2010	8	65	28	84	8.3	0.37 [0.18, 0.76]
Dambrink, 2012	27	79	15	40	11.1	0.91 [0.55, 1.51]
DANAMI 3, 2015	17	313	52	314	10.8	0.33 [0.19, 0.55]
Zhang, 2015	27	215	62	213	12.4	0.43 [0.29, 0.65]
Complete, 2019	29	2016	160	2025	12.7	0.18 [0.12, 0.27]
Random effects model for immediate studies ($p < 0.001$)						0.38 [0.23, 0.65]
$Q = 25.63$, df = 4, p for heterogeneity = 0.00; $I^2 = 82.8\%$						

Evidence of an immediate versus staged moderating effect: p = 0.86

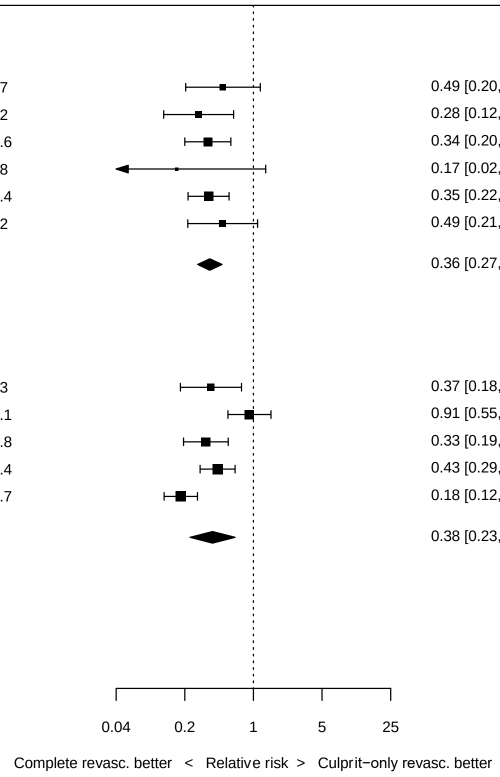

Figure S6. Effect of FFR-guided revascularization on cardiovascular death.

Study and Year	Active Events	Active N	Control Events	Control N	Weight (%)	Relative risk [95% CI]
FFR−guided revascularisation − risk of CV death						
DANAMI 3, 2015	5	313	9	314	9.8	0.56 [0.19, 1.64]
Compare ACUTE, 2017	3	295	6	590	6.4	1.00 [0.25, 3.97]
Random effects model for FFR−guided studies (p = 0.405)						0.70 [0.30, 1.63]
Q = 0.43, df = 1, p for heterogeneity = 0.51; I^2 = 0.0%						
Angiograpgy−guided revascularisation − risk of CV death						
Politi, 2010	6	130	10	84	11.7	0.39 [0.15, 1.03]
PRAMI, 2013	4	234	10	231	8.9	0.39 [0.13, 1.24]
Zhang, 2015	11	215	14	213	17	0.78 [0.36, 1.68]
Complete, 2019	59	2016	64	2025	41.2	0.93 [0.65, 1.31]
CvLPRIT, 2019	2	150	7	146	5.1	0.28 [0.06, 1.32]
Random effects model for angiography−guided studies (p = 0.055)						0.64 [0.40, 1.01]
Q = 5.93, df = 4, p for heterogeneity = 0.20; I^2 = 37.6%						

Evidence of an FFR−guided versus angiography−guided moderating effect: p = 0.73

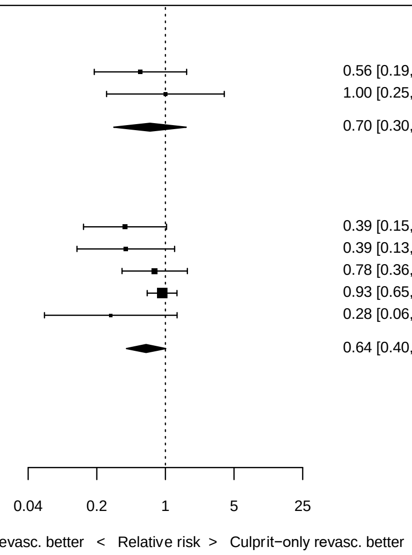

Figure S7. Effect of FFR-guided revascularization on all-cause mortality.

Study and Year	Active Events	Active N	Control Events	Control N	Weight (%)	Relative risk [95% CI]
FFR−guided revascularisation − risk of death						
Dambrink, 2012	2	79	0	40	0.5	2.56 [0.13, 52.14]
DANAMI 3, 2015	15	313	11	314	7.2	1.37 [0.64, 2.93]
Compare ACUTE, 2017	4	295	10	590	3.2	0.80 [0.25, 2.53]
Random effects model for FFR−guided studies (p = 0.563)						1.20 [0.65, 2.24]
Q = 0.83, df = 2, p for heterogeneity = 0.66; I^2 = 0.0%						
Angiograpgy−guided revascularisation − risk of death						
Help−AMI, 2009	1	52	0	17	0.4	1.02 [0.04, 23.91]
Politi, 2010	10	130	13	84	7	0.50 [0.23, 1.08]
PRAMI, 2013	12	234	16	231	8	0.74 [0.36, 1.53]
Zhang, 2015	13	215	15	213	8.2	0.86 [0.42, 1.76]
Hamza, 2016	1	50	4	50	0.9	0.25 [0.03, 2.16]
Complete, 2019	96	2016	106	2025	58	0.91 [0.70, 1.19]
CvLPRIT, 2019	9	150	15	146	6.7	0.58 [0.26, 1.29]
Random effects model for angiography−guided studies (p = 0.059)						0.79 [0.62, 1.01]
Q = 4.13, df = 6, p for heterogeneity = 0.66; I^2 = 5.6%						

Evidence of an FFR−guided versus angiography−guided moderating effect: p = 0.54

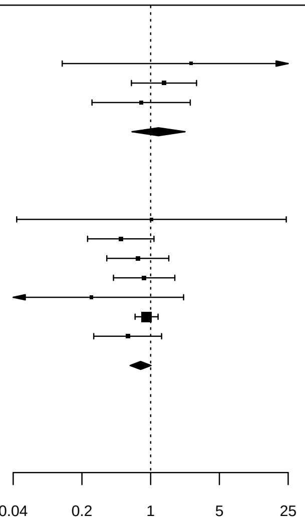

Complete revasc. better < Relative risk > Culprit−only revasc. better

Figure S8. Effect of FFR-guided revascularization on myocardial infarction.

Study and Year	Active Events	Active N	Control Events	Control N	Weight (%)	Relative risk [95% CI]
FFR−guided revascularisation − risk of MI						
Dambrink, 2012	4	79	0	40	0.4	4.61 [0.25, 83.61]
DANAMI 3, 2015	15	313	16	314	7.9	0.94 [0.47, 1.87]
Compare ACUTE, 2017	7	295	28	590	5.6	0.50 [0.22, 1.13]
Random effects model for FFR−guided studies (p = 0.399)						0.77 [0.42, 1.41]
Q = 2.87, df = 2, p for heterogeneity = 0.24; I^2 = 16.0%						
Angiograpgy−guided revascularisation − risk of MI						
Help−AMI, 2009	1	52	1	17	0.5	0.33 [0.02, 4.95]
Politi, 2010	6	130	7	84	3.3	0.55 [0.19, 1.59]
PRAMI, 2013	7	234	20	231	5.2	0.35 [0.15, 0.80]
Zhang, 2015	9	215	14	213	5.6	0.64 [0.28, 1.44]
Hamza, 2016	1	50	2	50	0.7	0.50 [0.05, 5.34]
Complete, 2019	109	2016	160	2025	66.8	0.68 [0.54, 0.87]
CvLPRIT, 2019	6	150	12	146	4.1	0.49 [0.19, 1.26]
Random effects model for angiography−guided studies (p = 0.000)						0.62 [0.48, 0.79]
Q = 3.03, df = 6, p for heterogeneity = 0.81; I^2 = 4.7%						

Evidence of an FFR−guided versus angiography−guided moderating effect: p = 0.68

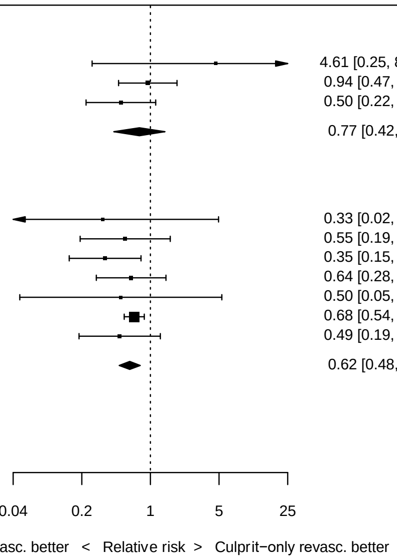

Figure S9. Effect of FFR-guided revascularization on unplanned revascularization.

Study and Year	Active Events	Active N	Control Events	Control N	Weight (%)	Relative risk [95% CI]
FFR−guided revascularisation − risk of unplanned revascularization						
Dambrink, 2012	27	79	15	40	11.7	0.91 [0.55, 1.51]
DANAMI 3, 2015	17	313	52	314	11.4	0.33 [0.19, 0.55]
Compare ACUTE, 2017	18	295	103	590	12	0.35 [0.22, 0.57]
Random effects model for FFR−guided studies (p = 0.023)						0.47 [0.25, 0.90]
Q = 9.90, df = 2, p for heterogeneity = 0.01; I^2 = 79.9%						
Angiograpgy−guided revascularisation − risk of unplanned revasc						
Help−AMI, 2009	9	52	6	17	7.2	0.49 [0.20, 1.18]
Politi, 2010	14	130	28	84	10.6	0.32 [0.18, 0.58]
PRAMI, 2013	16	234	46	231	11.2	0.34 [0.20, 0.59]
Zhang, 2015	27	215	62	213	13	0.43 [0.29, 0.65]
Hamza, 2016	1	50	6	50	1.9	0.17 [0.02, 1.33]
Complete, 2019	29	2016	160	2025	13.3	0.18 [0.12, 0.27]
CvLPRIT, 2019	8	150	16	146	7.7	0.49 [0.21, 1.10]
Random effects model for angiography−guided studies (p = 0.000)						0.33 [0.24, 0.46]
Q = 12.34, df = 6, p for heterogeneity = 0.05; I^2 = 50.4%						

Evidence of an FFR−guided versus angiography−guided moderating effect: p = 0.79

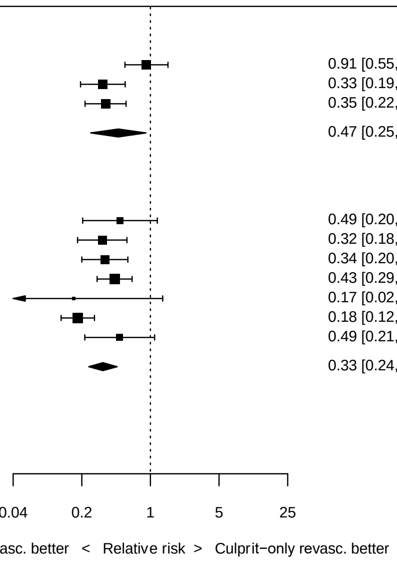

Figure S10. Freedom from cardiovascular death.

Study and Year	Active Events	Active N	Control Events	Control N	Weight (%)	Hazard ratio [95% CI]
Freedom from cardiovascular death						
CvLPRIT, 2019	2	150	7	146	8.8	0.27 [0.06, 1.32]
Complete, 2019	59	2016	64	2025	49.6	0.93 [0.66, 1.32]
Compare ACUTE, 2017	3	295	6	590	11	1.00 [0.25, 4.01]
DANAMI 3, 2015	5	313	9	314	15.8	0.56 [0.18, 1.70]
PRAMI, 2013	4	234	10	231	14.8	0.34 [0.11, 1.08]
RE Model for All Studies (Q = 5.11, df = 4, p for heterogeneity = 0.28; I^2 = 29.4%)						0.67 [0.40, 1.11]

p for overall effect = 0.119

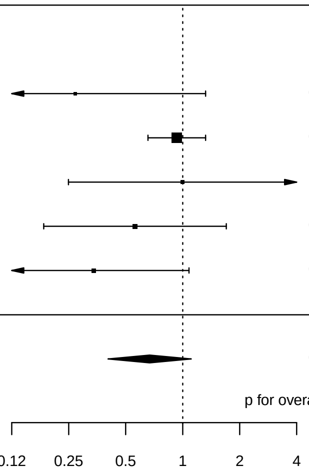

Complete revasc. better < Hazard ratio > Culprit−only revasc. better

Figure S11. Freedom from myocardial infarction.

Study and Year	Active Events	Active N	Control Events	Control N	Weight (%)	Hazard ratio [95% CI]
Freedom from MI						
CvLPRIT, 2019	6	150	12	146	6.2	0.43 [0.16, 1.15]
Complete, 2019	109	2016	160	2025	64.9	0.68 [0.54, 0.86]
Compare ACUTE, 2017	7	295	28	590	8.9	0.50 [0.22, 1.13]
DANAMI 3, 2015	15	313	16	314	11.7	0.94 [0.47, 1.90]
PRAMI, 2013	7	234	20	231	8.2	0.32 [0.14, 0.75]
RE Model for All Studies (Q = 4.92, df = 4, p for heterogeneity = 0.30; I^2 = 9.5%)						0.63 [0.49, 0.81]

p for overall effect < 0.001

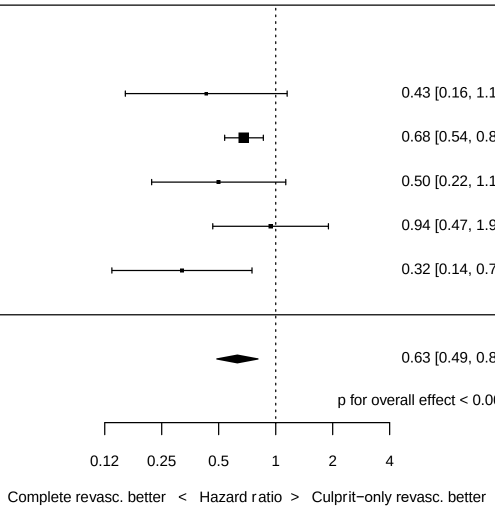

Figure S12. Freedom from all-cause death.

Study and Year	Active Events	Active N	Control Events	Control N	Weight (%)	Hazard ratio [95% CI]
Freedom from death						
CvLPRIT, 2019	9	150	15	146	7.9	0.51 [0.22, 1.16]
Complete, 2019	96	2016	106	2025	70.1	0.91 [0.69, 1.20]
Compare ACUTE, 2017	4	295	10	590	4	0.80 [0.25, 2.56]
DANAMI 3, 2015	15	313	11	314	9.2	1.40 [0.65, 3.00]
PRAMI, 2013	12	234	16	231	8.8	0.64 [0.29, 1.40]
RE Model for All Studies (Q = 3.83, df = 4, p for heterogeneity = 0.43; I^2 = 0.0%)						0.87 [0.69, 1.10]

p for overall effect = 0.248

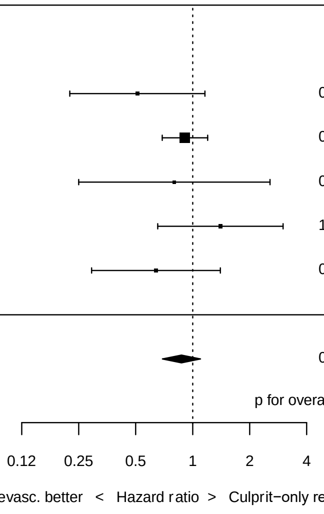

Figure S13. Freedom from unplanned revascularization.

Study and Year	Active Events	Active N	Control Events	Control N	Weight (%)	Hazard ratio [95% CI]
Freedom from unplanned revascularisation						
CvLPRIT, 2019	8	150	16	146	11	0.46 [0.20, 1.08]
Complete, 2019	29	2016	160	2025	29.8	0.18 [0.12, 0.26]
Compare ACUTE, 2017	18	295	103	590	21.3	0.32 [0.19, 0.54]
DANAMI 3, 2015	17	313	52	314	20.7	0.31 [0.18, 0.53]
PRAMI, 2013	16	234	46	231	17.2	0.30 [0.16, 0.56]
RE Model for All Studies (Q = 6.77, df = 4, p for heterogeneity = 0.15; I^2 = 42.1%)						0.28 [0.20, 0.38]

p for overall effect < 0.001

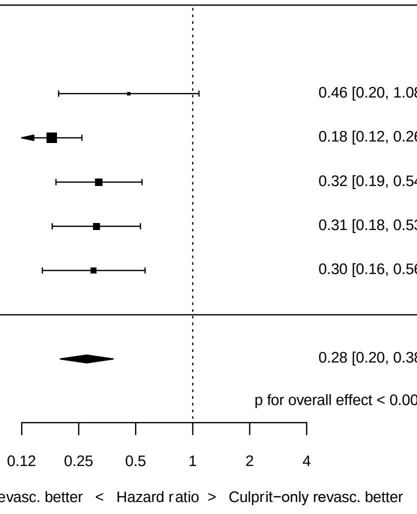

Figure S14. Fixed effects analysis for effect of complete revascularization on risk of cardiovascular death.

Study and Year	Active Events	Active N	Control Events	Control N	Weight (%)	Relative risk [95% CI]
Risk of cardiovascular death						
CvLPRIT, 2019	2	150	7	146	3.1	0.28 [0.06, 1.32]
Complete, 2019	59	2016	64	2025	60.8	0.93 [0.65, 1.31]
Compare ACUTE, 2017	3	295	6	590	3.9	1.00 [0.25, 3.97]
Zhang, 2015	11	215	14	213	12.6	0.78 [0.36, 1.68]
DANAMI 3, 2015	5	313	9	314	6.3	0.56 [0.19, 1.64]
PRAMI, 2013	4	234	10	231	5.6	0.39 [0.13, 1.24]
Politi, 2010	6	130	10	84	7.8	0.39 [0.15, 1.03]
FE Model for All Studies (Q = 6.40, df = 6, p for heterogeneity = 0.38; I^2 = 6.3%)						0.76 [0.58, 0.99]

p for overall effect = 0.043

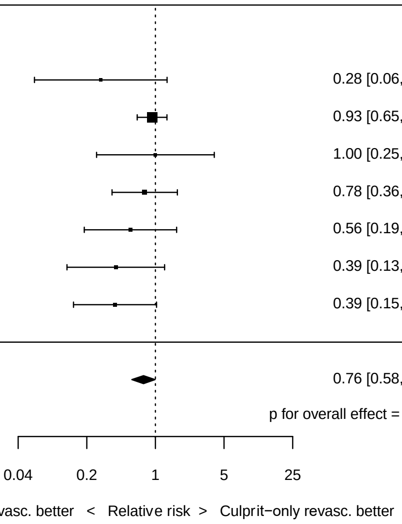

Figure S15. Fixed effects analysis for effect of complete revascularization on risk of myocardial infarction.

Study and Year	Active Events	Active N	Control Events	Control N	Weight (%)	Relative risk [95% CI]
Risk of MI						
CvLPRIT, 2019	6	150	12	146	4.1	0.49 [0.19, 1.26]
Complete, 2019	109	2016	160	2025	66.8	0.68 [0.54, 0.87]
Compare ACUTE, 2017	7	295	28	590	5.6	0.50 [0.22, 1.13]
Hamza, 2016	1	50	2	50	0.7	0.50 [0.05, 5.34]
Zhang, 2015	9	215	14	213	5.6	0.64 [0.28, 1.44]
DANAMI 3, 2015	15	313	16	314	7.9	0.94 [0.47, 1.87]
PRAMI, 2013	7	234	20	231	5.2	0.35 [0.15, 0.80]
Dambrink, 2012	4	79	0	40	0.4	4.61 [0.25, 83.61]
Politi, 2010	6	130	7	84	3.3	0.55 [0.19, 1.59]
Help−AMI, 2009	1	52	1	17	0.5	0.33 [0.02, 4.95]
FE Model for All Studies (Q = 6.35, df = 9, p for heterogeneity = 0.70; I^2 = 0.0%)						0.65 [0.54, 0.79]
						p for overall effect < 0.001

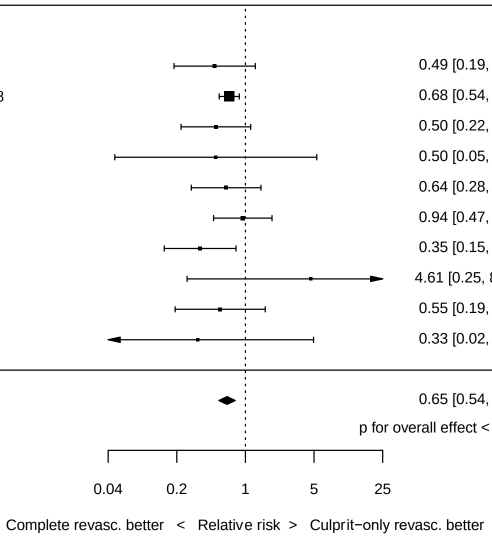

Figure S16. Fixed effects analysis for effect of complete revascularization on risk of all-cause mortality.

Study and Year	Active Events	Active N	Control Events	Control N	Weight (%)	Relative risk [95% CI]
Risk of death						
CvLPRIT, 2019	9	150	15	146	6.7	0.58 [0.26, 1.29]
Complete, 2019	96	2016	106	2025	58	0.91 [0.70, 1.19]
Compare ACUTE, 2017	4	295	10	590	3.2	0.80 [0.25, 2.53]
Hamza, 2016	1	50	4	50	0.9	0.25 [0.03, 2.16]
Zhang, 2015	13	215	15	213	8.2	0.86 [0.42, 1.76]
DANAMI 3, 2015	15	313	11	314	7.2	1.37 [0.64, 2.93]
PRAMI, 2013	12	234	16	231	8	0.74 [0.36, 1.53]
Dambrink, 2012	2	79	0	40	0.5	2.56 [0.13, 52.14]
Politi, 2010	10	130	13	84	7	0.50 [0.23, 1.08]
Help−AMI, 2009	1	52	0	17	0.4	1.02 [0.04, 23.91]
FE Model for All Studies (Q = 6.34, df = 9, p for heterogeneity = 0.71; I^2 = 0.0%)						0.85 [0.69, 1.04]
						p for overall effect = 0.108

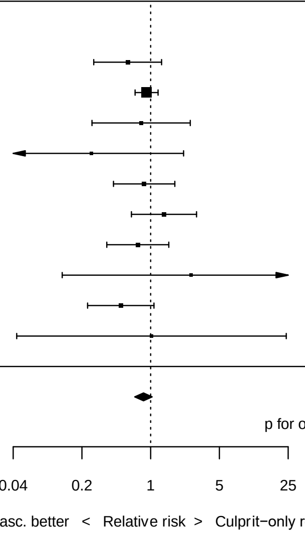

Figure S17. Fixed effects analysis for effect of complete revascularization on risk of unplanned revascularization.

Study and Year	Active Events	Active N	Control Events	Control N	Weight (%)	Relative risk [95% CI]
Risk of unplanned revascularisation						
CvLPRIT, 2019	8	150	16	146	4.4	0.49 [0.21, 1.10]
Complete, 2019	29	2016	160	2025	19.4	0.18 [0.12, 0.27]
Compare ACUTE, 2017	18	295	103	590	12.8	0.35 [0.22, 0.57]
Hamza, 2016	1	50	6	50	0.7	0.17 [0.02, 1.33]
Zhang, 2015	27	215	62	213	17.6	0.43 [0.29, 0.65]
DANAMI 3, 2015	17	313	52	314	10.7	0.33 [0.19, 0.55]
PRAMI, 2013	16	234	46	231	10.2	0.34 [0.20, 0.59]
Dambrink, 2012	27	79	15	40	11.6	0.91 [0.55, 1.51]
Politi, 2010	14	130	28	84	8.8	0.32 [0.18, 0.58]
Help−AMI, 2009	9	52	6	17	3.9	0.49 [0.20, 1.18]
FE Model for All Studies (Q = 27.33, df = 9, p for heterogeneity = 0.00; I^2 = 67.1%)						0.36 [0.30, 0.43]
						p for overall effect < 0.001

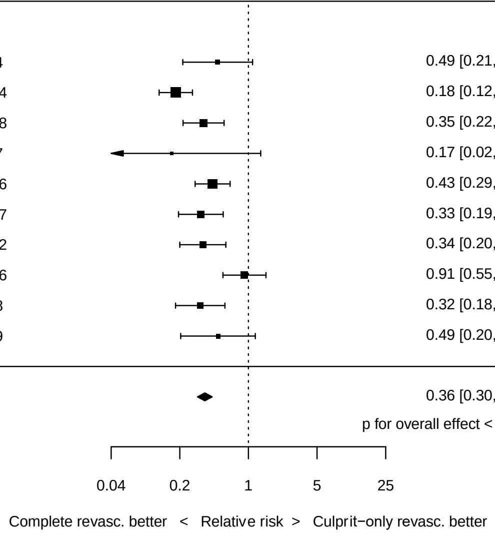

Figure S18. Fixed effects analysis for effect of complete revascularization on risk of major bleeding.

Study and Year	Active Events	Active N	Control Events	Control N	Weight (%)	Relative risk [95% CI]
Risk of bleeding						
CvLPRIT, 2019	4	150	7	146	7.4	0.56 [0.17, 1.86]
Complete, 2019	58	2016	44	2025	71.8	1.32 [0.90, 1.95]
Compare ACUTE, 2017	3	295	8	590	6.2	0.75 [0.20, 2.81]
Hamza, 2016	0	50	0	50	0.7	1.00 [0.02, 49.44]
DANAMI 3, 2015	1	313	4	314	2.3	0.25 [0.03, 2.23]
PRAMI, 2013	7	234	6	231	9.3	1.15 [0.39, 3.38]
Dambrink, 2012	5	79	1	40	2.4	2.53 [0.31, 20.95]
FE Model for All Studies (Q = 4.71, df = 6, p for heterogeneity = 0.58; I^2 = 0.0%)						1.16 [0.83, 1.60]

p for overall effect = 0.387

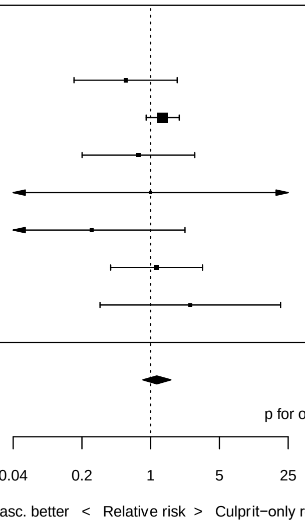

Figure S19. Sensitivity analysis for risk of cardiovascular death including only trials at low-risk of bias.

Study and Year	Active Events	Active N	Control Events	Control N	Weight (%)	Relative risk [95% CI]
Risk of cardiovascular death						
CvLPRIT, 2019	2	150	7	146	7.5	0.28 [0.06, 1.32]
Complete, 2019	59	2016	64	2025	41.4	0.93 [0.65, 1.31]
Compare ACUTE, 2017	3	295	6	590	9.2	1.00 [0.25, 3.97]
DANAMI 3, 2015	5	313	9	314	13.6	0.56 [0.19, 1.64]
PRAMI, 2013	4	234	10	231	12.4	0.39 [0.13, 1.24]
Politi, 2010	6	130	10	84	15.8	0.39 [0.15, 1.03]
RE Model for All Studies (Q = 6.39, df = 5, p for heterogeneity = 0.27; I^2 = 31.4%)						0.62 [0.39, 0.99]

p for overall effect = 0.044

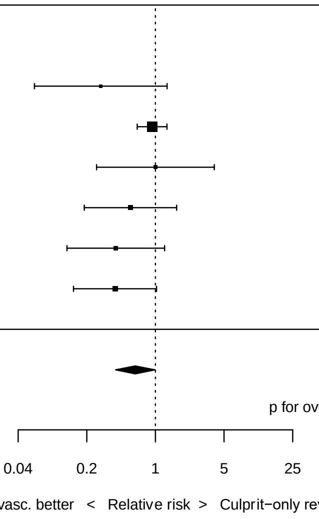

Figure S20. Sensitivity analysis for risk of myocardial infarction including only trials at low-risk of bias.

Study and Year	Active Events	Active N	Control Events	Control N	Weight (%)	Relative risk [95% CI]
Risk of MI						
CvLPRIT, 2019	6	150	12	146	4.4	0.49 [0.19, 1.26]
Complete, 2019	109	2016	160	2025	71.6	0.68 [0.54, 0.87]
Compare ACUTE, 2017	7	295	28	590	6	0.50 [0.22, 1.13]
DANAMI 3, 2015	15	313	16	314	8.4	0.94 [0.47, 1.87]
PRAMI, 2013	7	234	20	231	5.6	0.35 [0.15, 0.80]
Dambrink, 2012	4	79	0	40	0.5	4.61 [0.25, 83.61]
Politi, 2010	6	130	7	84	3.6	0.55 [0.19, 1.59]
RE Model for All Studies (Q = 6.05, df = 6, p for heterogeneity = 0.42; I^2 = 0.0%)						0.66 [0.54, 0.80]
						p for overall effect < 0.001

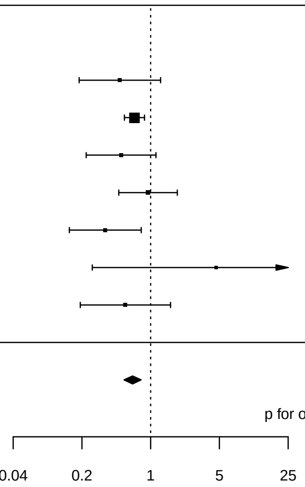

Figure S21. Sensitivity analysis for risk of all-cause mortality including only trials at low-risk of bias.

Study and Year	Active Events	Active N	Control Events	Control N	Weight (%)	Relative risk [95% CI]
Risk of death						
CvLPRIT, 2019	9	150	15	146	7.4	0.58 [0.26, 1.29]
Complete, 2019	96	2016	106	2025	64.1	0.91 [0.70, 1.19]
Compare ACUTE, 2017	4	295	10	590	3.5	0.80 [0.25, 2.53]
DANAMI 3, 2015	15	313	11	314	8	1.37 [0.64, 2.93]
PRAMI, 2013	12	234	16	231	8.8	0.74 [0.36, 1.53]
Dambrink, 2012	2	79	0	40	0.5	2.56 [0.13, 52.14]
Politi, 2010	10	130	13	84	7.7	0.50 [0.23, 1.08]
RE Model for All Studies (Q = 5.09, df = 6, p for heterogeneity = 0.53; I^2 = 0.0%)						0.85 [0.69, 1.06]

p for overall effect = 0.150

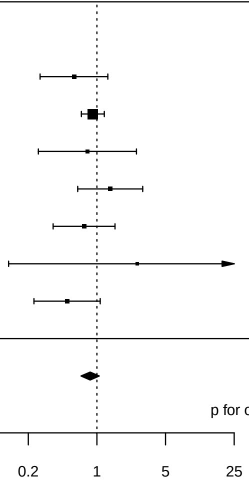

Complete revasc. better < Relative risk > Culprit−only revasc. better

Figure S22. Sensitivity analysis for risk of unplanned revascularization including only trials at low-risk of bias.

Study and Year	Active Events	Active N	Control Events	Control N	Weight (%)	Relative risk [95% CI]
Risk of unplanned revascularisation						
CvLPRIT, 2019	8	150	16	146	10.6	0.49 [0.21, 1.10]
Complete, 2019	29	2016	160	2025	16.6	0.18 [0.12, 0.27]
Compare ACUTE, 2017	18	295	103	590	15.2	0.35 [0.22, 0.57]
DANAMI 3, 2015	17	313	52	314	14.6	0.33 [0.19, 0.55]
PRAMI, 2013	16	234	46	231	14.4	0.34 [0.20, 0.59]
Dambrink, 2012	27	79	15	40	14.9	0.91 [0.55, 1.51]
Politi, 2010	14	130	28	84	13.8	0.32 [0.18, 0.58]
RE Model for All Studies (Q = 25.33, df = 6, p for heterogeneity = 0.00; I^2 = 73.5%)						0.37 [0.25, 0.54]

p for overall effect < 0.001

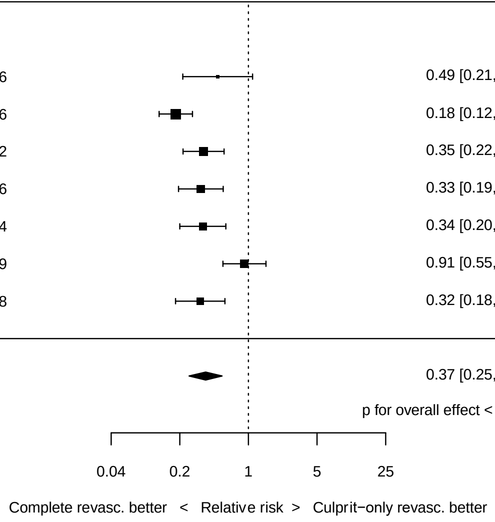

Figure S23. Sensitivity analysis excluding trials with low use of drug-eluting stents for the effect of complete revascularization on risk of cardiovascular death.

Study and Year	Active Events	Active N	Control Events	Control N	Weight (%)	Relative risk [95% CI]
Risk of cardiovascular death						
CvLPRIT, 2019	2	150	7	146	4.2	0.28 [0.06, 1.32]
Complete, 2019	59	2016	64	2025	58.4	0.93 [0.65, 1.31]
Compare ACUTE, 2017	3	295	6	590	5.3	1.00 [0.25, 3.97]
Zhang, 2015	11	215	14	213	16.1	0.78 [0.36, 1.68]
DANAMI 3, 2015	5	313	9	314	8.5	0.56 [0.19, 1.64]
PRAMI, 2013	4	234	10	231	7.6	0.39 [0.13, 1.24]
RE Model for All Studies (Q = 4.45, df = 5, p for heterogeneity = 0.49; I^2 = 7.0%)						0.77 [0.56, 1.07]

p for overall effect = 0.115

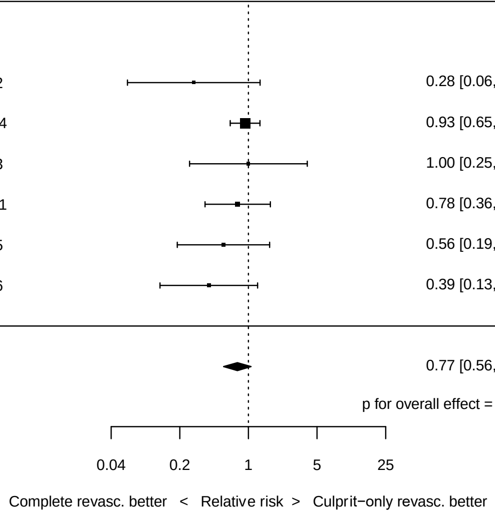

Figure S24. Sensitivity analysis excluding trials with low use of drug-eluting stents for the effect of complete revascularization on risk of myocardial infarction.

Study and Year	Active Events	Active N	Control Events	Control N	Weight (%)	Relative risk [95% CI]
Risk of MI						
CvLPRIT, 2019	6	150	12	146	4.3	0.49 [0.19, 1.26]
Complete, 2019	109	2016	160	2025	69.8	0.68 [0.54, 0.87]
Compare ACUTE, 2017	7	295	28	590	5.8	0.50 [0.22, 1.13]
Hamza, 2016	1	50	2	50	0.7	0.50 [0.05, 5.34]
Zhang, 2015	9	215	14	213	5.8	0.64 [0.28, 1.44]
DANAMI 3, 2015	15	313	16	314	8.2	0.94 [0.47, 1.87]
PRAMI, 2013	7	234	20	231	5.5	0.35 [0.15, 0.80]
RE Model for All Studies (Q = 4.26, df = 6, p for heterogeneity = 0.64; I^2 = 0.0%)						0.65 [0.53, 0.79]

p for overall effect < 0.001

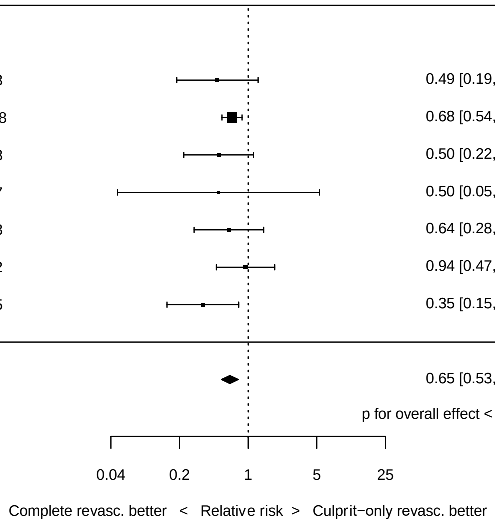

Figure S25. Sensitivity analysis excluding trials with low use of drug-eluting stents for the effect of complete revascularization on risk of all-cause mortality.

Study and Year	Active Events	Active N	Control Events	Control N	Weight (%)	Relative risk [95% CI]
Risk of death						
CvLPRIT, 2019	9	150	15	146	7.2	0.58 [0.26, 1.29]
Complete, 2019	96	2016	106	2025	63	0.91 [0.70, 1.19]
Compare ACUTE, 2017	4	295	10	590	3.4	0.80 [0.25, 2.53]
Hamza, 2016	1	50	4	50	1	0.25 [0.03, 2.16]
Zhang, 2015	13	215	15	213	8.8	0.86 [0.42, 1.76]
DANAMI 3, 2015	15	313	11	314	7.9	1.37 [0.64, 2.93]
PRAMI, 2013	12	234	16	231	8.7	0.74 [0.36, 1.53]
RE Model for All Studies (Q = 3.92, df = 6, p for heterogeneity = 0.69; I^2 = 0.0%)						0.87 [0.71, 1.08]

p for overall effect = 0.217

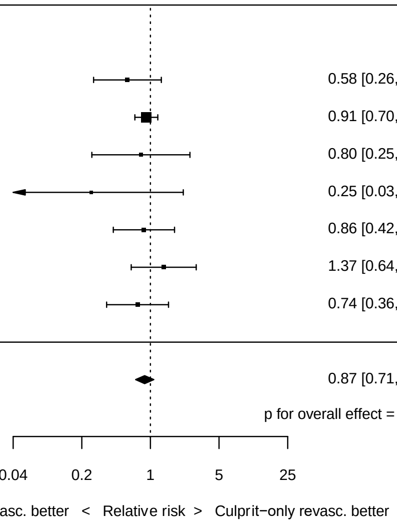

Figure S26. Sensitivity analysis excluding trials with low use of drug-eluting stents for the effect of complete revascularization on risk of unplanned revascularization.

Study and Year	Active Events	Active N	Control Events	Control N	Weight (%)	Relative risk [95% CI]
Risk of unplanned revascularisation						
CvLPRIT, 2019	8	150	16	146	9.3	0.49 [0.21, 1.10]
Complete, 2019	29	2016	160	2025	20.5	0.18 [0.12, 0.27]
Compare ACUTE, 2017	18	295	103	590	17.3	0.35 [0.22, 0.57]
Hamza, 2016	1	50	6	50	1.9	0.17 [0.02, 1.33]
Zhang, 2015	27	215	62	213	19.7	0.43 [0.29, 0.65]
DANAMI 3, 2015	17	313	52	314	15.9	0.33 [0.19, 0.55]
PRAMI, 2013	16	234	46	231	15.5	0.34 [0.20, 0.59]
RE Model for All Studies (Q = 11.55, df = 6, p for heterogeneity = 0.07; I^2 = 48.5%)						0.32 [0.24, 0.43]

p for overall effect < 0.001

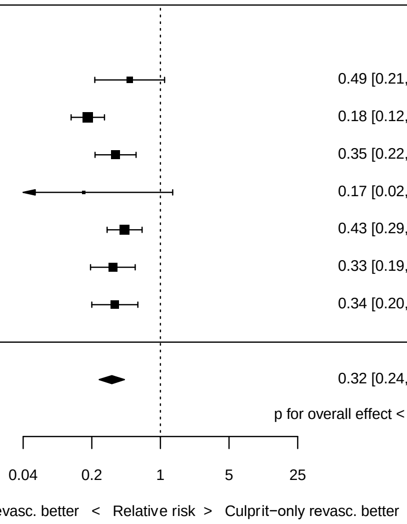

Figure S27. Sensitivity analysis excluding trials with low use of drug-eluting stents for the effect of complete revascularization on risk of major bleeding.

Study and Year	Active Events	Active N	Control Events	Control N	Weight (%)	Relative risk [95% CI]
Risk of bleeding						
CvLPRIT, 2019	4	150	7	146	11.8	0.56 [0.17, 1.86]
Complete, 2019	58	2016	44	2025	58.5	1.32 [0.90, 1.95]
Compare ACUTE, 2017	3	295	8	590	10.1	0.75 [0.20, 2.81]
Hamza, 2016	0	50	0	50	1.3	1.00 [0.02, 49.44]
DANAMI 3, 2015	1	313	4	314	3.9	0.25 [0.03, 2.23]
PRAMI, 2013	7	234	6	231	14.5	1.15 [0.39, 3.38]
RE Model for All Studies (Q = 4.17, df = 5, p for heterogeneity = 0.53; I^2 = 12.6%)						1.03 [0.67, 1.60]

p for overall effect = 0.885

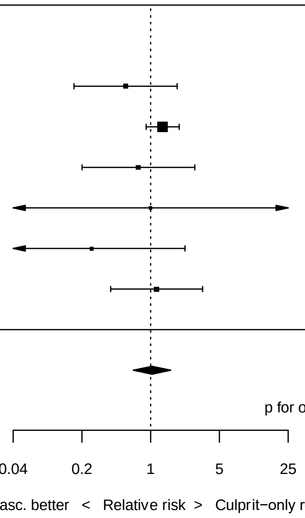

Figure S28. Sensitivity analysis for risk of cardiovascular death excluding the COMPARE ACUTE trial.

Study and Year	Active Events	Active N	Control Events	Control N	Weight (%)	Relative risk [95% CI]
Risk of CV death						
CvLPRIT, 2019	2	150	7	146	6.1	0.28 [0.06, 1.32]
Complete, 2019	59	2016	64	2025	39.9	0.93 [0.65, 1.31]
Zhang, 2015	11	215	14	213	18.8	0.78 [0.36, 1.68]
DANAMI 3, 2015	5	313	9	314	11.4	0.56 [0.19, 1.64]
PRAMI, 2013	4	234	10	231	10.4	0.39 [0.13, 1.24]
Politi, 2010	6	130	10	84	13.4	0.39 [0.15, 1.03]
RE Model for All Studies (Q = 6.24, df = 5, p for heterogeneity = 0.28; I^2 = 30.4%)						0.64 [0.43, 0.96]

p for overall effect = 0.032

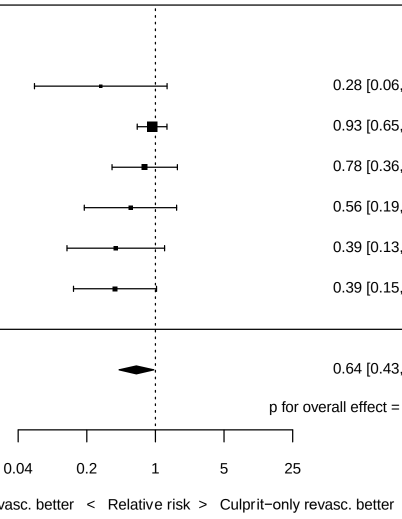

Figure S29. Sensitivity analysis for risk of cardiovascular death excluding the COMPLETE trial.

Study and Year	Active Events	Active N	Control Events	Control N	Weight (%)	Relative risk [95% CI]
Risk of CV death						
CvLPRIT, 2019	2	150	7	146	7.8	0.28 [0.06, 1.32]
Compare ACUTE, 2017	3	295	6	590	9.9	1.00 [0.25, 3.97]
Zhang, 2015	11	215	14	213	32	0.78 [0.36, 1.68]
DANAMI 3, 2015	5	313	9	314	16.1	0.56 [0.19, 1.64]
PRAMI, 2013	4	234	10	231	14.4	0.39 [0.13, 1.24]
Politi, 2010	6	130	10	84	19.8	0.39 [0.15, 1.03]
RE Model for All Studies ($Q = 3.07$, $df = 5$, p for heterogeneity = 0.69; $I^2 = 0.0\%$)						0.55 [0.36, 0.85]

p for overall effect = 0.007

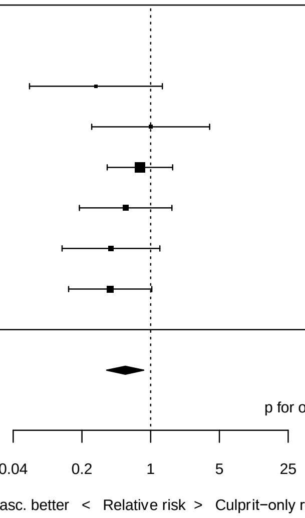

Figure S30. Sensitivity analysis for risk of cardiovascular death excluding the CVLPRIT trial

Study and Year	Active Events	Active N	Control Events	Control N	Weight (%)	Relative risk [95% CI]
Risk of CV death						
Complete, 2019	59	2016	64	2025	48.2	0.93 [0.65, 1.31]
Compare ACUTE, 2017	3	295	6	590	6	1.00 [0.25, 3.97]
Zhang, 2015	11	215	14	213	16.9	0.78 [0.36, 1.68]
DANAMI 3, 2015	5	313	9	314	9.3	0.56 [0.19, 1.64]
PRAMI, 2013	4	234	10	231	8.4	0.39 [0.13, 1.24]
Politi, 2010	6	130	10	84	11.2	0.39 [0.15, 1.03]
RE Model for All Studies (Q = 4.76, df = 5, p for heterogeneity = 0.45; I^2 = 16.3%)						0.73 [0.51, 1.03]

p for overall effect = 0.073

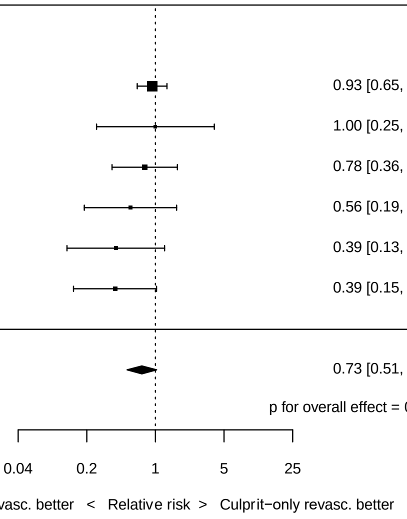

Figure S31. Sensitivity analysis for risk of cardiovascular death excluding the DANAMI 3 trial

Study and Year	Active Events	Active N	Control Events	Control N	Weight (%)	Relative risk [95% CI]
Risk of CV death						
CvLPRIT, 2019	2	150	7	146	6.1	0.28 [0.06, 1.32]
Complete, 2019	59	2016	64	2025	43.4	0.93 [0.65, 1.31]
Compare ACUTE, 2017	3	295	6	590	7.5	1.00 [0.25, 3.97]
Zhang, 2015	11	215	14	213	19.3	0.78 [0.36, 1.68]
PRAMI, 2013	4	234	10	231	10.3	0.39 [0.13, 1.24]
Politi, 2010	6	130	10	84	13.5	0.39 [0.15, 1.03]
RE Model for All Studies (Q = 6.08, df = 5, p for heterogeneity = 0.30; I^2 = 25.9%)						0.68 [0.46, 1.02]

p for overall effect = 0.062

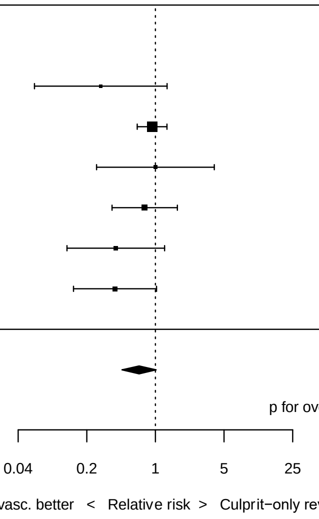

Figure S32. Sensitivity analysis for risk of cardiovascular death excluding the Politi trial

Study and Year	Active Events	Active N	Control Events	Control N	Weight (%)	Relative risk [95% CI]
Risk of CV death						
CvLPRIT, 2019	2	150	7	146	4.2	0.28 [0.06, 1.32]
Complete, 2019	59	2016	64	2025	58.4	0.93 [0.65, 1.31]
Compare ACUTE, 2017	3	295	6	590	5.3	1.00 [0.25, 3.97]
Zhang, 2015	11	215	14	213	16.1	0.78 [0.36, 1.68]
DANAMI 3, 2015	5	313	9	314	8.5	0.56 [0.19, 1.64]
PRAMI, 2013	4	234	10	231	7.6	0.39 [0.13, 1.24]
RE Model for All Studies (Q = 4.45, df = 5, p for heterogeneity = 0.49; I^2 = 7.0%)						0.77 [0.56, 1.07]

p for overall effect = 0.115

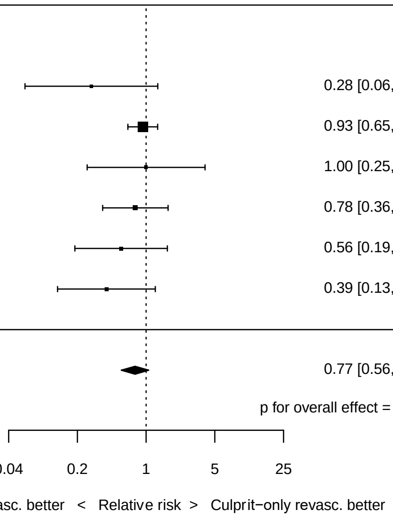

Figure S33. Sensitivity analysis for risk of cardiovascular death excluding the PRAMI trial

Study and Year	Active Events	Active N	Control Events	Control N	Weight (%)	Relative risk [95% CI]
Risk of CV death						
CvLPRIT, 2019	2	150	7	146	5	0.28 [0.06, 1.32]
Complete, 2019	59	2016	64	2025	49.7	0.93 [0.65, 1.31]
Compare ACUTE, 2017	3	295	6	590	6.3	1.00 [0.25, 3.97]
Zhang, 2015	11	215	14	213	17.6	0.78 [0.36, 1.68]
DANAMI 3, 2015	5	313	9	314	9.8	0.56 [0.19, 1.64]
Politi, 2010	6	130	10	84	11.7	0.39 [0.15, 1.03]
RE Model for All Studies (Q = 5.09, df = 5, p for heterogeneity = 0.40; I^2 = 16.0%)						0.73 [0.51, 1.04]

p for overall effect = 0.084

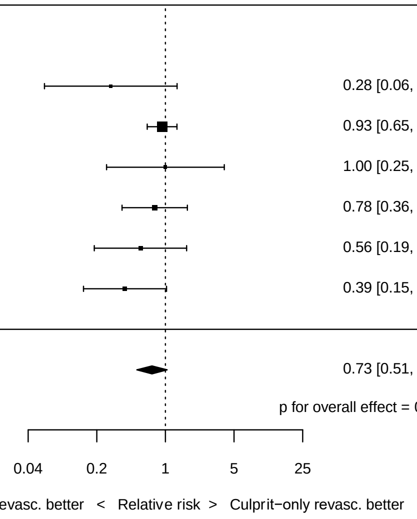

Figure S34. Sensitivity analysis for risk of cardiovascular death excluding the Zhang trial

Study and Year	Active Events	Active N	Control Events	Control N	Weight (%)	Relative risk [95% CI]
Risk of CV death						
CvLPRIT, 2019	2	150	7	146	7.5	0.28 [0.06, 1.32]
Complete, 2019	59	2016	64	2025	41.4	0.93 [0.65, 1.31]
Compare ACUTE, 2017	3	295	6	590	9.2	1.00 [0.25, 3.97]
DANAMI 3, 2015	5	313	9	314	13.6	0.56 [0.19, 1.64]
PRAMI, 2013	4	234	10	231	12.4	0.39 [0.13, 1.24]
Politi, 2010	6	130	10	84	15.8	0.39 [0.15, 1.03]
RE Model for All Studies (Q = 6.39, df = 5, p for heterogeneity = 0.27; I^2 = 31.4%)						0.62 [0.39, 0.99]

p for overall effect = 0.044

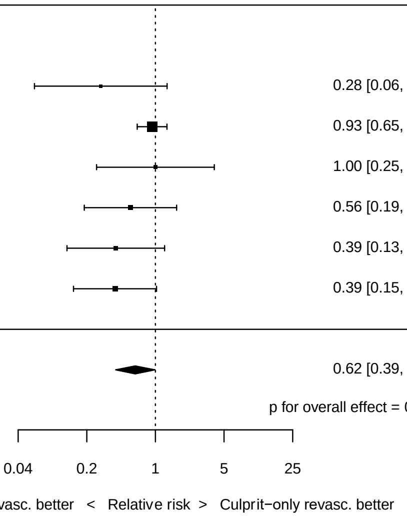

Figure S35. Sensitivity analysis for risk of myocardial infarction excluding the COMPARE ACUTE trial

Study and Year	Active Events	Active N	Control Events	Control N	Weight (%)	Relative risk [95% CI]
Risk of MI						
CvLPRIT, 2019	6	150	12	146	4.3	0.49 [0.19, 1.26]
Complete, 2019	109	2016	160	2025	70.7	0.68 [0.54, 0.87]
Hamza, 2016	1	50	2	50	0.7	0.50 [0.05, 5.34]
Zhang, 2015	9	215	14	213	5.9	0.64 [0.28, 1.44]
DANAMI 3, 2015	15	313	16	314	8.3	0.94 [0.47, 1.87]
PRAMI, 2013	7	234	20	231	5.5	0.35 [0.15, 0.80]
Dambrink, 2012	4	79	0	40	0.5	4.61 [0.25, 83.61]
Politi, 2010	6	130	7	84	3.5	0.55 [0.19, 1.59]
Help−AMI, 2009	1	52	1	17	0.5	0.33 [0.02, 4.95]
RE Model for All Studies (Q = 5.93, df = 8, p for heterogeneity = 0.66; I^2 = 0.0%)						0.66 [0.54, 0.81]

p for overall effect < 0.001

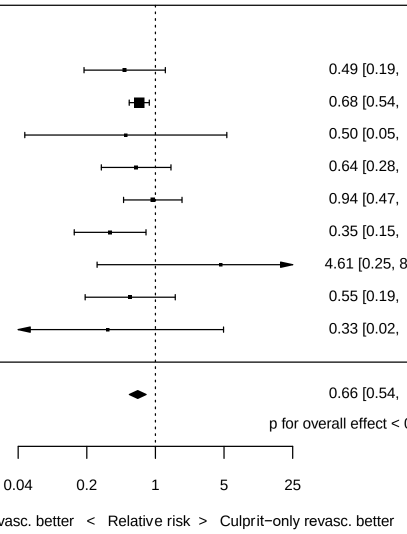

Figure 36. Sensitivity analysis for risk of myocardial infarction excluding the COMPLETE trial

Study and Year	Active Events	Active N	Control Events	Control N	Weight (%)	Relative risk [95% CI]
Risk of MI						
CvLPRIT, 2019	6	150	12	146	12.3	0.49 [0.19, 1.26]
Compare ACUTE, 2017	7	295	28	590	16.7	0.50 [0.22, 1.13]
Hamza, 2016	1	50	2	50	2	0.50 [0.05, 5.34]
Zhang, 2015	9	215	14	213	16.8	0.64 [0.28, 1.44]
DANAMI 3, 2015	15	313	16	314	23.6	0.94 [0.47, 1.87]
PRAMI, 2013	7	234	20	231	15.8	0.35 [0.15, 0.80]
Dambrink, 2012	4	79	0	40	1.3	4.61 [0.25, 83.61]
Politi, 2010	6	130	7	84	10	0.55 [0.19, 1.59]
Help−AMI, 2009	1	52	1	17	1.5	0.33 [0.02, 4.95]
RE Model for All Studies (Q = 5.82, df = 8, p for heterogeneity = 0.67; I^2 = 0.0%)						0.59 [0.42, 0.82]

p for overall effect = 0.002

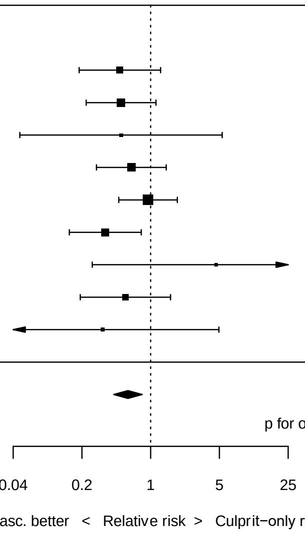

Figure S37. Sensitivity analysis for risk of myocardial infarction excluding the CvLPRIT trial

Study and Year	Active Events	Active N	Control Events	Control N	Weight (%)	Relative risk [95% CI]
Risk of MI						
Complete, 2019	109	2016	160	2025	69.6	0.68 [0.54, 0.87]
Compare ACUTE, 2017	7	295	28	590	5.8	0.50 [0.22, 1.13]
Hamza, 2016	1	50	2	50	0.7	0.50 [0.05, 5.34]
Zhang, 2015	9	215	14	213	5.8	0.64 [0.28, 1.44]
DANAMI 3, 2015	15	313	16	314	8.2	0.94 [0.47, 1.87]
PRAMI, 2013	7	234	20	231	5.5	0.35 [0.15, 0.80]
Dambrink, 2012	4	79	0	40	0.5	4.61 [0.25, 83.61]
Politi, 2010	6	130	7	84	3.5	0.55 [0.19, 1.59]
Help−AMI, 2009	1	52	1	17	0.5	0.33 [0.02, 4.95]
RE Model for All Studies (Q = 5.98, df = 8, p for heterogeneity = 0.65; I^2 = 0.0%)						0.66 [0.54, 0.80]

p for overall effect < 0.001

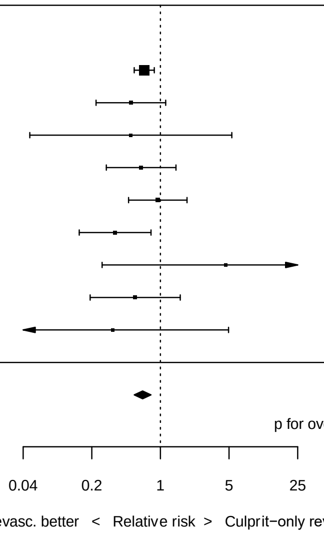

Figure S38. Sensitivity analysis for risk of myocardial infarction excluding the Dambrink trial

Study and Year	Active Events	Active N	Control Events	Control N	Weight (%)	Relative risk [95% CI]
Risk of MI						
CvLPRIT, 2019	6	150	12	146	4.1	0.49 [0.19, 1.26]
Complete, 2019	109	2016	160	2025	67.1	0.68 [0.54, 0.87]
Compare ACUTE, 2017	7	295	28	590	5.6	0.50 [0.22, 1.13]
Hamza, 2016	1	50	2	50	0.7	0.50 [0.05, 5.34]
Zhang, 2015	9	215	14	213	5.6	0.64 [0.28, 1.44]
DANAMI 3, 2015	15	313	16	314	7.9	0.94 [0.47, 1.87]
PRAMI, 2013	7	234	20	231	5.3	0.35 [0.15, 0.80]
Politi, 2010	6	130	7	84	3.3	0.55 [0.19, 1.59]
Help−AMI, 2009	1	52	1	17	0.5	0.33 [0.02, 4.95]
RE Model for All Studies (Q = 4.59, df = 8, p for heterogeneity = 0.80; I^2 = 0.0%)						0.64 [0.53, 0.78]

p for overall effect < 0.001

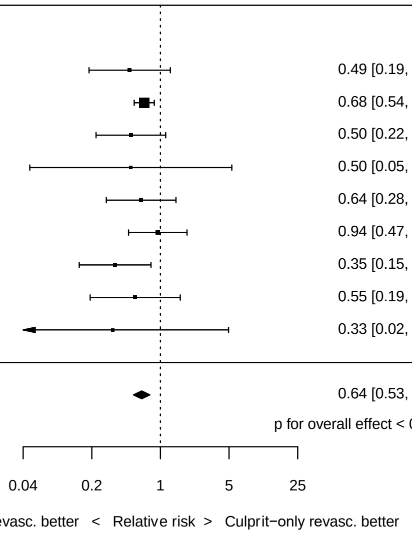

Figure S39. Sensitivity analysis for risk of myocardial infarction excluding the DANAMI 3 trial

Study and Year	Active Events	Active N	Control Events	Control N	Weight (%)	Relative risk [95% CI]
Risk of MI						
CvLPRIT, 2019	6	150	12	146	4.9	0.49 [0.19, 1.26]
Complete, 2019	109	2016	160	2025	69.3	0.68 [0.54, 0.87]
Compare ACUTE, 2017	7	295	28	590	6.7	0.50 [0.22, 1.13]
Hamza, 2016	1	50	2	50	0.8	0.50 [0.05, 5.34]
Zhang, 2015	9	215	14	213	6.7	0.64 [0.28, 1.44]
PRAMI, 2013	7	234	20	231	6.3	0.35 [0.15, 0.80]
Dambrink, 2012	4	79	0	40	0.5	4.61 [0.25, 83.61]
Politi, 2010	6	130	7	84	4	0.55 [0.19, 1.59]
Help−AMI, 2009	1	52	1	17	0.6	0.33 [0.02, 4.95]
RE Model for All Studies (Q = 5.15, df = 8, p for heterogeneity = 0.74; I^2 = 1.4%)						0.62 [0.50, 0.77]
						p for overall effect < 0.001

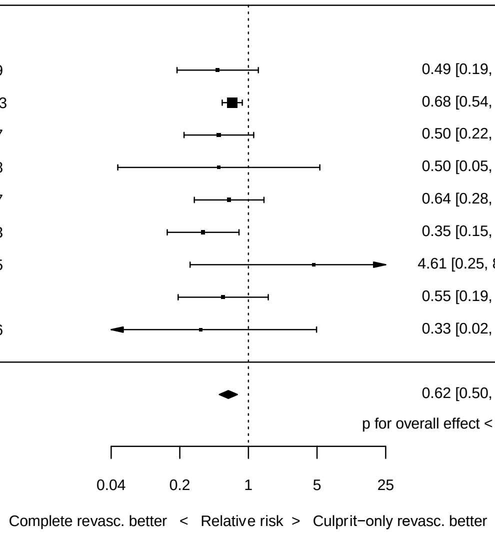

Figure S40. Sensitivity analysis for risk of myocardial infarction excluding the Hamza trial

Study and Year	Active Events	Active N	Control Events	Control N	Weight (%)	Relative risk [95% CI]
Risk of MI						
CvLPRIT, 2019	6	150	12	146	4.1	0.49 [0.19, 1.26]
Complete, 2019	109	2016	160	2025	67.2	0.68 [0.54, 0.87]
Compare ACUTE, 2017	7	295	28	590	5.6	0.50 [0.22, 1.13]
Zhang, 2015	9	215	14	213	5.6	0.64 [0.28, 1.44]
DANAMI 3, 2015	15	313	16	314	7.9	0.94 [0.47, 1.87]
PRAMI, 2013	7	234	20	231	5.3	0.35 [0.15, 0.80]
Dambrink, 2012	4	79	0	40	0.4	4.61 [0.25, 83.61]
Politi, 2010	6	130	7	84	3.3	0.55 [0.19, 1.59]
Help−AMI, 2009	1	52	1	17	0.5	0.33 [0.02, 4.95]
RE Model for All Studies (Q = 6.31, df = 8, p for heterogeneity = 0.61; I^2 = 0.0%)						0.65 [0.54, 0.79]
						p for overall effect < 0.001

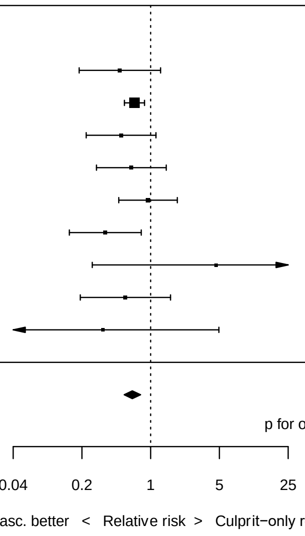

Figure S41. Sensitivity analysis for risk of myocardial infarction excluding the HELP-AMI trial

Study and Year	Active Events	Active N	Control Events	Control N	Weight (%)	Relative risk [95% CI]
Risk of MI						
CvLPRIT, 2019	6	150	12	146	4.1	0.49 [0.19, 1.26]
Complete, 2019	109	2016	160	2025	67.1	0.68 [0.54, 0.87]
Compare ACUTE, 2017	7	295	28	590	5.6	0.50 [0.22, 1.13]
Hamza, 2016	1	50	2	50	0.7	0.50 [0.05, 5.34]
Zhang, 2015	9	215	14	213	5.6	0.64 [0.28, 1.44]
DANAMI 3, 2015	15	313	16	314	7.9	0.94 [0.47, 1.87]
PRAMI, 2013	7	234	20	231	5.3	0.35 [0.15, 0.80]
Dambrink, 2012	4	79	0	40	0.4	4.61 [0.25, 83.61]
Politi, 2010	6	130	7	84	3.3	0.55 [0.19, 1.59]
RE Model for All Studies (Q = 6.11, df = 8, p for heterogeneity = 0.64; I^2 = 0.0%)						0.65 [0.54, 0.79]

p for overall effect < 0.001

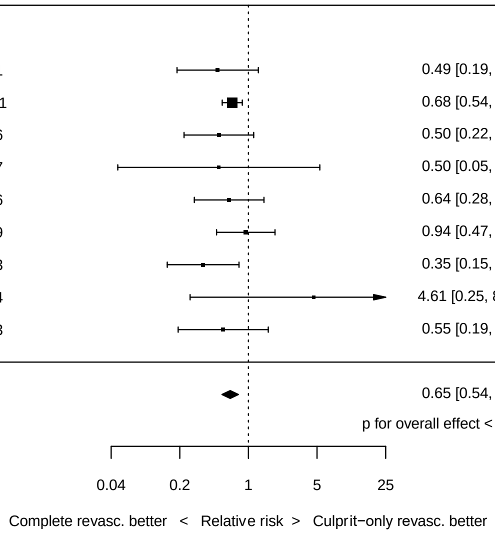

Figure S42. Sensitivity analysis for risk of myocardial infarction excluding the Politi trial

Study and Year	Active Events	Active N	Control Events	Control N	Weight (%)	Relative risk [95% CI]
Risk of MI						
CvLPRIT, 2019	6	150	12	146	4.2	0.49 [0.19, 1.26]
Complete, 2019	109	2016	160	2025	69.1	0.68 [0.54, 0.87]
Compare ACUTE, 2017	7	295	28	590	5.7	0.50 [0.22, 1.13]
Hamza, 2016	1	50	2	50	0.7	0.50 [0.05, 5.34]
Zhang, 2015	9	215	14	213	5.8	0.64 [0.28, 1.44]
DANAMI 3, 2015	15	313	16	314	8.1	0.94 [0.47, 1.87]
PRAMI, 2013	7	234	20	231	5.4	0.35 [0.15, 0.80]
Dambrink, 2012	4	79	0	40	0.5	4.61 [0.25, 83.61]
Help−AMI, 2009	1	52	1	17	0.5	0.33 [0.02, 4.95]
RE Model for All Studies (Q = 6.26, df = 8, p for heterogeneity = 0.62; I^2 = 0.0%)						0.65 [0.54, 0.80]

p for overall effect < 0.001

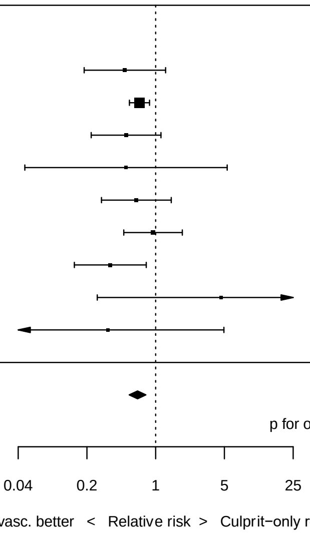

Figure S43. Sensitivity analysis for risk of myocardial infarction excluding the PRAMI trial

Study and Year	Active Events	Active N	Control Events	Control N	Weight (%)	Relative risk [95% CI]
Risk of MI						
CvLPRIT, 2019	6	150	12	146	4.3	0.49 [0.19, 1.26]
Complete, 2019	109	2016	160	2025	70.5	0.68 [0.54, 0.87]
Compare ACUTE, 2017	7	295	28	590	5.9	0.50 [0.22, 1.13]
Hamza, 2016	1	50	2	50	0.7	0.50 [0.05, 5.34]
Zhang, 2015	9	215	14	213	5.9	0.64 [0.28, 1.44]
DANAMI 3, 2015	15	313	16	314	8.3	0.94 [0.47, 1.87]
Dambrink, 2012	4	79	0	40	0.5	4.61 [0.25, 83.61]
Politi, 2010	6	130	7	84	3.5	0.55 [0.19, 1.59]
Help−AMI, 2009	1	52	1	17	0.5	0.33 [0.02, 4.95]
RE Model for All Studies (Q = 4.06, df = 8, p for heterogeneity = 0.85; I^2 = 0.0%)						0.67 [0.55, 0.82]

p for overall effect < 0.001

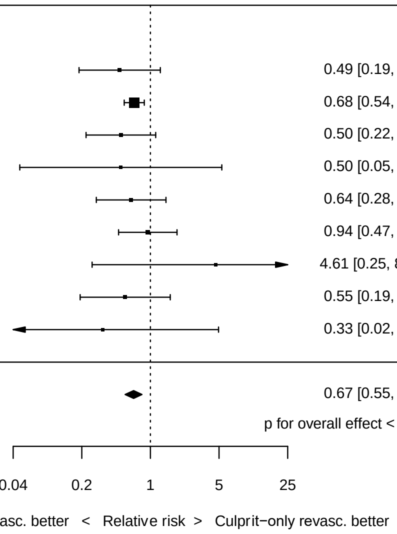

Figure S44. Sensitivity analysis for risk of myocardial infarction excluding the Zhang trial

Study and Year	Active Events	Active N	Control Events	Control N	Weight (%)	Relative risk [95% CI]
Risk of MI						
CvLPRIT, 2019	6	150	12	146	4.3	0.49 [0.19, 1.26]
Complete, 2019	109	2016	160	2025	70.7	0.68 [0.54, 0.87]
Compare ACUTE, 2017	7	295	28	590	5.9	0.50 [0.22, 1.13]
Hamza, 2016	1	50	2	50	0.7	0.50 [0.05, 5.34]
DANAMI 3, 2015	15	313	16	314	8.3	0.94 [0.47, 1.87]
PRAMI, 2013	7	234	20	231	5.5	0.35 [0.15, 0.80]
Dambrink, 2012	4	79	0	40	0.5	4.61 [0.25, 83.61]
Politi, 2010	6	130	7	84	3.5	0.55 [0.19, 1.59]
Help−AMI, 2009	1	52	1	17	0.5	0.33 [0.02, 4.95]
RE Model for All Studies (Q = 6.35, df = 8, p for heterogeneity = 0.61; I^2 = 0.0%)						0.65 [0.53, 0.79]

p for overall effect < 0.001

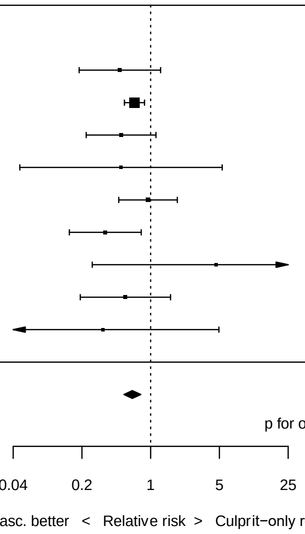

Figure S45. Sensitivity analysis for risk of all-cause mortality excluding the COMPARE ACUTE trial

Study and Year	Active Events	Active N	Control Events	Control N	Weight (%)	Relative risk [95% CI]
Risk of death						
CvLPRIT, 2019	9	150	15	146	6.9	0.58 [0.26, 1.29]
Complete, 2019	96	2016	106	2025	59.9	0.91 [0.70, 1.19]
Hamza, 2016	1	50	4	50	0.9	0.25 [0.03, 2.16]
Zhang, 2015	13	215	15	213	8.4	0.86 [0.42, 1.76]
DANAMI 3, 2015	15	313	11	314	7.5	1.37 [0.64, 2.93]
PRAMI, 2013	12	234	16	231	8.2	0.74 [0.36, 1.53]
Dambrink, 2012	2	79	0	40	0.5	2.56 [0.13, 52.14]
Politi, 2010	10	130	13	84	7.2	0.50 [0.23, 1.08]
Help−AMI, 2009	1	52	0	17	0.4	1.02 [0.04, 23.91]
RE Model for All Studies (Q = 6.33, df = 8, p for heterogeneity = 0.61; I^2 = 0.0%)						0.85 [0.69, 1.04]

p for overall effect = 0.117

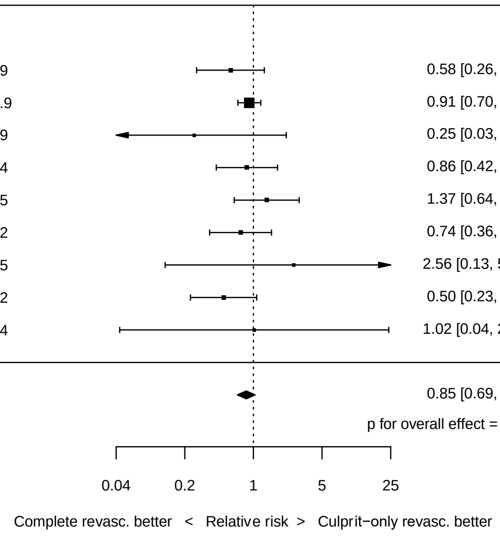

Figure S46. Sensitivity analysis for risk of all-cause mortality excluding the COMPLETE trial

Study and Year	Active Events	Active N	Control Events	Control N	Weight (%)	Relative risk [95% CI]
Risk of death						
CvLPRIT, 2019	9	150	15	146	15.9	0.58 [0.26, 1.29]
Compare ACUTE, 2017	4	295	10	590	7.6	0.80 [0.25, 2.53]
Hamza, 2016	1	50	4	50	2.2	0.25 [0.03, 2.16]
Zhang, 2015	13	215	15	213	19.4	0.86 [0.42, 1.76]
DANAMI 3, 2015	15	313	11	314	17.3	1.37 [0.64, 2.93]
PRAMI, 2013	12	234	16	231	19	0.74 [0.36, 1.53]
Dambrink, 2012	2	79	0	40	1.1	2.56 [0.13, 52.14]
Politi, 2010	10	130	13	84	16.6	0.50 [0.23, 1.08]
Help−AMI, 2009	1	52	0	17	1	1.02 [0.04, 23.91]
RE Model for All Studies (Q = 5.66, df = 8, p for heterogeneity = 0.69; I^2 = 0.0%)						0.76 [0.56, 1.05]
						p for overall effect = 0.094

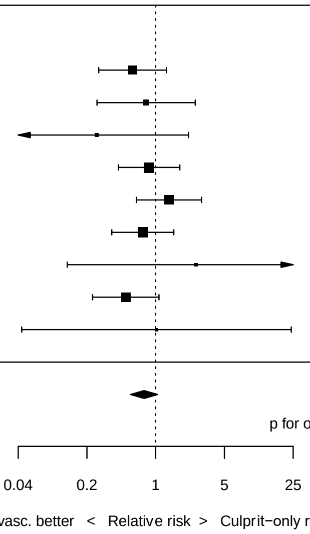

Figure S47. Sensitivity analysis for risk of all-cause mortality excluding the CvLPRIT trial

Study and Year	Active Events	Active N	Control Events	Control N	Weight (%)	Relative risk [95% CI]
Risk of death						
Complete, 2019	96	2016	106	2025	62.2	0.91 [0.70, 1.19]
Compare ACUTE, 2017	4	295	10	590	3.4	0.80 [0.25, 2.53]
Hamza, 2016	1	50	4	50	1	0.25 [0.03, 2.16]
Zhang, 2015	13	215	15	213	8.7	0.86 [0.42, 1.76]
DANAMI 3, 2015	15	313	11	314	7.8	1.37 [0.64, 2.93]
PRAMI, 2013	12	234	16	231	8.5	0.74 [0.36, 1.53]
Dambrink, 2012	2	79	0	40	0.5	2.56 [0.13, 52.14]
Politi, 2010	10	130	13	84	7.5	0.50 [0.23, 1.08]
Help−AMI, 2009	1	52	0	17	0.5	1.02 [0.04, 23.91]
RE Model for All Studies (Q = 5.45, df = 8, p for heterogeneity = 0.71; I^2 = 0.0%)						0.87 [0.70, 1.07]

p for overall effect = 0.190

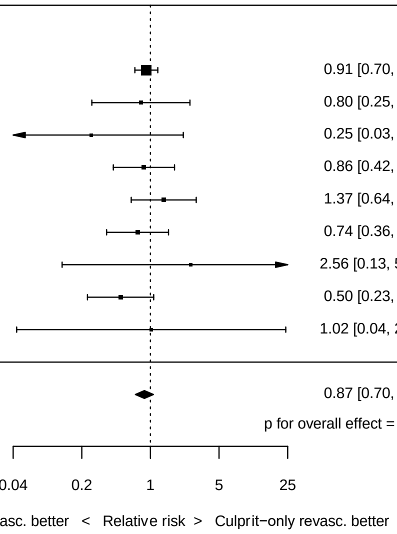

Figure S48. Sensitivity analysis for risk of all-cause mortality excluding the Dambrink trial

Study and Year	Active Events	Active N	Control Events	Control N	Weight (%)	Relative risk [95% CI]
Risk of death						
CvLPRIT, 2019	9	150	15	146	6.7	0.58 [0.26, 1.29]
Complete, 2019	96	2016	106	2025	58.3	0.91 [0.70, 1.19]
Compare ACUTE, 2017	4	295	10	590	3.2	0.80 [0.25, 2.53]
Hamza, 2016	1	50	4	50	0.9	0.25 [0.03, 2.16]
Zhang, 2015	13	215	15	213	8.2	0.86 [0.42, 1.76]
DANAMI 3, 2015	15	313	11	314	7.3	1.37 [0.64, 2.93]
PRAMI, 2013	12	234	16	231	8	0.74 [0.36, 1.53]
Politi, 2010	10	130	13	84	7	0.50 [0.23, 1.08]
Help−AMI, 2009	1	52	0	17	0.4	1.02 [0.04, 23.91]
RE Model for All Studies (Q = 5.82, df = 8, p for heterogeneity = 0.67; I^2 = 0.0%)						0.84 [0.68, 1.03]

p for overall effect = 0.098

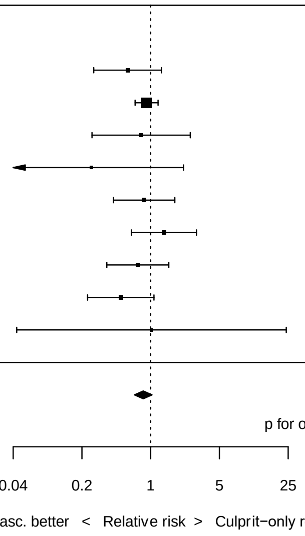

Figure S49. Sensitivity analysis for risk of all-cause mortality excluding the DANAMI 3 trial

Study and Year	Active Events	Active N	Control Events	Control N	Weight (%)	Relative risk [95% CI]
Risk of death						
CvLPRIT, 2019	9	150	15	146	7.4	0.58 [0.26, 1.29]
Complete, 2019	96	2016	106	2025	61.5	0.91 [0.70, 1.19]
Compare ACUTE, 2017	4	295	10	590	3.5	0.80 [0.25, 2.53]
Hamza, 2016	1	50	4	50	1	0.25 [0.03, 2.16]
Zhang, 2015	13	215	15	213	9	0.86 [0.42, 1.76]
PRAMI, 2013	12	234	16	231	8.8	0.74 [0.36, 1.53]
Dambrink, 2012	2	79	0	40	0.5	2.56 [0.13, 52.14]
Politi, 2010	10	130	13	84	7.7	0.50 [0.23, 1.08]
Help−AMI, 2009	1	52	0	17	0.5	1.02 [0.04, 23.91]
RE Model for All Studies (Q = 4.69, df = 8, p for heterogeneity = 0.79; I^2 = 0.6%)						0.81 [0.65, 1.01]

p for overall effect = 0.058

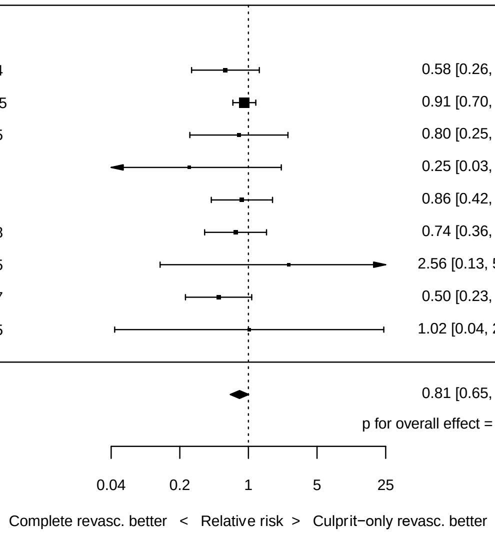

Figure S50. Sensitivity analysis for risk of all-cause mortality excluding the Hamza trial

Study and Year	Active		Control		Weight (%)	Relative risk [95% CI]
	Events	N	Events	N		
Risk of death						
CvLPRIT, 2019	9	150	15	146	6.7	0.58 [0.26, 1.29]
Complete, 2019	96	2016	106	2025	58.6	0.91 [0.70, 1.19]
Compare ACUTE, 2017	4	295	10	590	3.2	0.80 [0.25, 2.53]
Zhang, 2015	13	215	15	213	8.2	0.86 [0.42, 1.76]
DANAMI 3, 2015	15	313	11	314	7.3	1.37 [0.64, 2.93]
PRAMI, 2013	12	234	16	231	8	0.74 [0.36, 1.53]
Dambrink, 2012	2	79	0	40	0.5	2.56 [0.13, 52.14]
Politi, 2010	10	130	13	84	7	0.50 [0.23, 1.08]
Help−AMI, 2009	1	52	0	17	0.4	1.02 [0.04, 23.91]
RE Model for All Studies (Q = 5.11, df = 8, p for heterogeneity = 0.75; I^2 = 0.0%)						0.85 [0.70, 1.05]

p for overall effect = 0.135

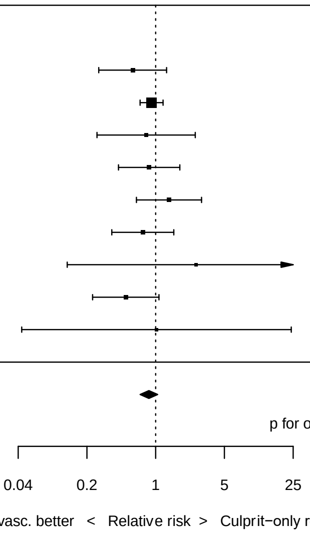

Figure S51. Sensitivity analysis for risk of all-cause mortality excluding the HELP-AMI trial

Study and Year	Active Events	Active N	Control Events	Control N	Weight (%)	Relative risk [95% CI]
Risk of death						
CvLPRIT, 2019	9	150	15	146	6.7	0.58 [0.26, 1.29]
Complete, 2019	96	2016	106	2025	58.3	0.91 [0.70, 1.19]
Compare ACUTE, 2017	4	295	10	590	3.2	0.80 [0.25, 2.53]
Hamza, 2016	1	50	4	50	0.9	0.25 [0.03, 2.16]
Zhang, 2015	13	215	15	213	8.2	0.86 [0.42, 1.76]
DANAMI 3, 2015	15	313	11	314	7.3	1.37 [0.64, 2.93]
PRAMI, 2013	12	234	16	231	8	0.74 [0.36, 1.53]
Dambrink, 2012	2	79	0	40	0.5	2.56 [0.13, 52.14]
Politi, 2010	10	130	13	84	7	0.50 [0.23, 1.08]
RE Model for All Studies (Q = 6.33, df = 8, p for heterogeneity = 0.61; I^2 = 0.0%)						0.84 [0.69, 1.04]
						p for overall effect = 0.107

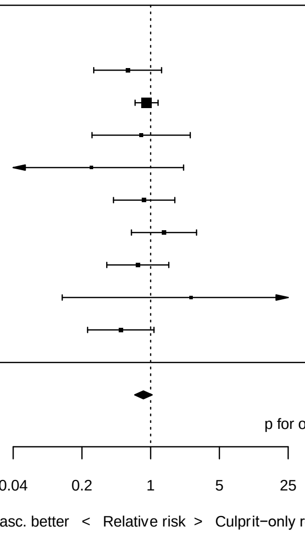

Figure S52. Sensitivity analysis for risk of all-cause mortality excluding the Politi trial

Study and Year	Active Events	Active N	Control Events	Control N	Weight (%)	Relative risk [95% CI]
Risk of death						
CvLPRIT, 2019	9	150	15	146	7.2	0.58 [0.26, 1.29]
Complete, 2019	96	2016	106	2025	62.4	0.91 [0.70, 1.19]
Compare ACUTE, 2017	4	295	10	590	3.4	0.80 [0.25, 2.53]
Hamza, 2016	1	50	4	50	1	0.25 [0.03, 2.16]
Zhang, 2015	13	215	15	213	8.8	0.86 [0.42, 1.76]
DANAMI 3, 2015	15	313	11	314	7.8	1.37 [0.64, 2.93]
PRAMI, 2013	12	234	16	231	8.6	0.74 [0.36, 1.53]
Dambrink, 2012	2	79	0	40	0.5	2.56 [0.13, 52.14]
Help−AMI, 2009	1	52	0	17	0.5	1.02 [0.04, 23.91]
RE Model for All Studies (Q = 4.42, df = 8, p for heterogeneity = 0.82; I^2 = 0.0%)						0.88 [0.71, 1.09]

p for overall effect = 0.236

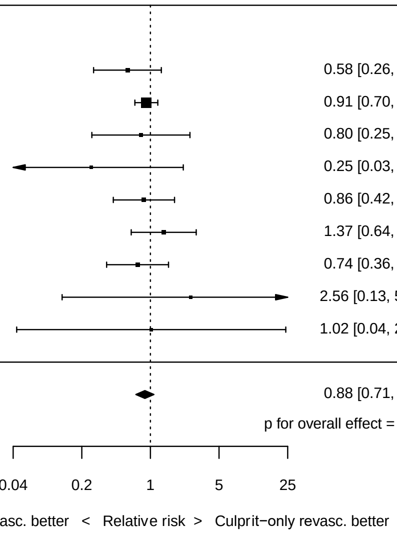

Figure S53. Sensitivity analysis for risk of all-cause mortality excluding the PRAMI trial

Study and Year	Active Events	Active N	Control Events	Control N	Weight (%)	Relative risk [95% CI]
Risk of death						
CvLPRIT, 2019	9	150	15	146	7.2	0.58 [0.26, 1.29]
Complete, 2019	96	2016	106	2025	63.1	0.91 [0.70, 1.19]
Compare ACUTE, 2017	4	295	10	590	3.4	0.80 [0.25, 2.53]
Hamza, 2016	1	50	4	50	1	0.25 [0.03, 2.16]
Zhang, 2015	13	215	15	213	8.9	0.86 [0.42, 1.76]
DANAMI 3, 2015	15	313	11	314	7.9	1.37 [0.64, 2.93]
Dambrink, 2012	2	79	0	40	0.5	2.56 [0.13, 52.14]
Politi, 2010	10	130	13	84	7.6	0.50 [0.23, 1.08]
Help−AMI, 2009	1	52	0	17	0.5	1.02 [0.04, 23.91]
RE Model for All Studies (Q = 6.20, df = 8, p for heterogeneity = 0.62; I^2 = 0.0%)						0.85 [0.69, 1.06]

p for overall effect = 0.150

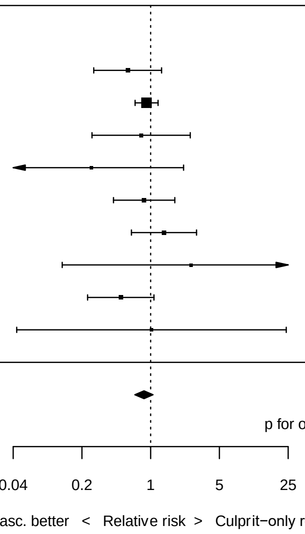

Figure S54. Sensitivity analysis for risk of all-cause mortality excluding the Zhang trial

Study and Year	Active Events	Active N	Control Events	Control N	Weight (%)	Relative risk [95% CI]
Risk of death						
CvLPRIT, 2019	9	150	15	146	7.3	0.58 [0.26, 1.29]
Complete, 2019	96	2016	106	2025	63.2	0.91 [0.70, 1.19]
Compare ACUTE, 2017	4	295	10	590	3.5	0.80 [0.25, 2.53]
Hamza, 2016	1	50	4	50	1	0.25 [0.03, 2.16]
DANAMI 3, 2015	15	313	11	314	7.9	1.37 [0.64, 2.93]
PRAMI, 2013	12	234	16	231	8.7	0.74 [0.36, 1.53]
Dambrink, 2012	2	79	0	40	0.5	2.56 [0.13, 52.14]
Politi, 2010	10	130	13	84	7.6	0.50 [0.23, 1.08]
Help−AMI, 2009	1	52	0	17	0.5	1.02 [0.04, 23.91]
RE Model for All Studies (Q = 6.34, df = 8, p for heterogeneity = 0.61; I^2 = 0.0%)						0.84 [0.68, 1.05]

p for overall effect = 0.120

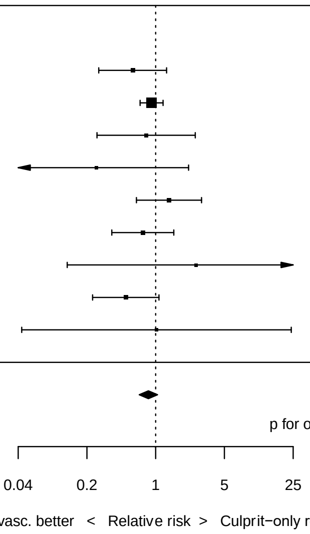

Figure S55. Sensitivity analysis for risk of unplanned revascularization excluding the COMPARE ACUTE trial

Study and Year	Active Events	Active N	Control Events	Control N	Weight (%)	Relative risk [95% CI]
Risk of unplanned revascularisation						
CvLPRIT, 2019	8	150	16	146	9.1	0.49 [0.21, 1.10]
Complete, 2019	29	2016	160	2025	14.8	0.18 [0.12, 0.27]
Hamza, 2016	1	50	6	50	2.4	0.17 [0.02, 1.33]
Zhang, 2015	27	215	62	213	14.5	0.43 [0.29, 0.65]
DANAMI 3, 2015	17	313	52	314	12.9	0.33 [0.19, 0.55]
PRAMI, 2013	16	234	46	231	12.7	0.34 [0.20, 0.59]
Dambrink, 2012	27	79	15	40	13.2	0.91 [0.55, 1.51]
Politi, 2010	14	130	28	84	12.1	0.32 [0.18, 0.58]
Help−AMI, 2009	9	52	6	17	8.5	0.49 [0.20, 1.18]
RE Model for All Studies (Q = 27.32, df = 8, p for heterogeneity = 0.00; I^2 = 67.7%)						0.38 [0.27, 0.54]

p for overall effect < 0.001

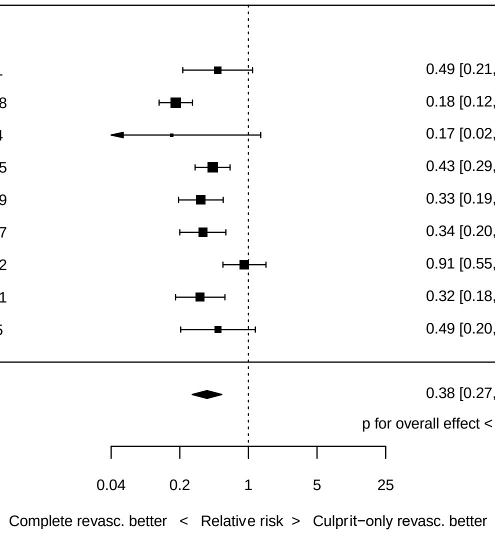

Figure S56. Sensitivity analysis for risk of unplanned revascularization excluding the COMPLETE trial

Study and Year	Active Events	Active N	Control Events	Control N	Weight (%)	Relative risk [95% CI]
Risk of unplanned revascularisation						
CvLPRIT, 2019	8	150	16	146	7.6	0.49 [0.21, 1.10]
Compare ACUTE, 2017	18	295	103	590	14.7	0.35 [0.22, 0.57]
Hamza, 2016	1	50	6	50	1.5	0.17 [0.02, 1.33]
Zhang, 2015	27	215	62	213	16.9	0.43 [0.29, 0.65]
DANAMI 3, 2015	17	313	52	314	13.4	0.33 [0.19, 0.55]
PRAMI, 2013	16	234	46	231	13	0.34 [0.20, 0.59]
Dambrink, 2012	27	79	15	40	14	0.91 [0.55, 1.51]
Politi, 2010	14	130	28	84	12	0.32 [0.18, 0.58]
Help−AMI, 2009	9	52	6	17	6.9	0.49 [0.20, 1.18]

RE Model for All Studies (Q = 12.84, df = 8, p for heterogeneity = 0.12; I^2 = 41.4%) 0.42 [0.32, 0.55]

p for overall effect < 0.001

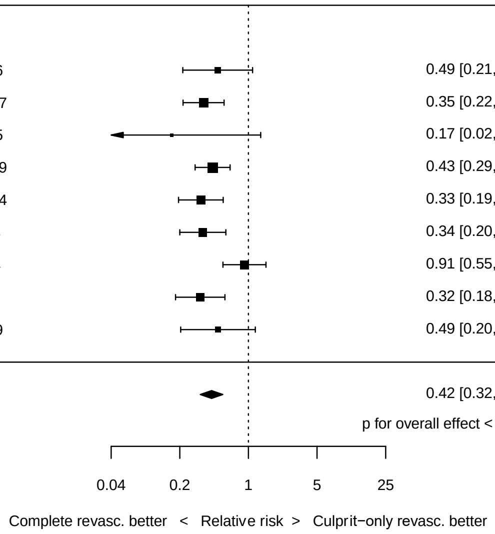

Figure S57. Sensitivity analysis for risk of unplanned revascularization excluding the CvLPRIT trial

Study and Year	Active Events	Active N	Control Events	Control N	Weight (%)	Relative risk [95% CI]
Risk of unplanned revascularisation						
Complete, 2019	29	2016	160	2025	14.3	0.18 [0.12, 0.27]
Compare ACUTE, 2017	18	295	103	590	13	0.35 [0.22, 0.57]
Hamza, 2016	1	50	6	50	2.2	0.17 [0.02, 1.33]
Zhang, 2015	27	215	62	213	14	0.43 [0.29, 0.65]
DANAMI 3, 2015	17	313	52	314	12.3	0.33 [0.19, 0.55]
PRAMI, 2013	16	234	46	231	12.1	0.34 [0.20, 0.59]
Dambrink, 2012	27	79	15	40	12.6	0.91 [0.55, 1.51]
Politi, 2010	14	130	28	84	11.5	0.32 [0.18, 0.58]
Help−AMI, 2009	9	52	6	17	7.9	0.49 [0.20, 1.18]
RE Model for All Studies (Q = 26.79, df = 8, p for heterogeneity = 0.00; I^2 = 68.1%)						0.37 [0.26, 0.51]

p for overall effect < 0.001

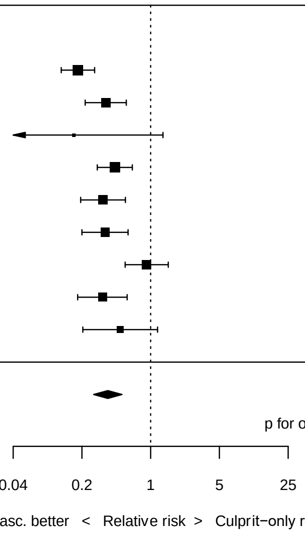

Figure S58. Sensitivity analysis for risk of unplanned revascularization excluding the Dambrink trial

Study and Year	Active Events	Active N	Control Events	Control N	Weight (%)	Relative risk [95% CI]
Risk of unplanned revascularisation						
CvLPRIT, 2019	8	150	16	146	7.1	0.49 [0.21, 1.10]
Complete, 2019	29	2016	160	2025	17.3	0.18 [0.12, 0.27]
Compare ACUTE, 2017	18	295	103	590	14.2	0.35 [0.22, 0.57]
Hamza, 2016	1	50	6	50	1.4	0.17 [0.02, 1.33]
Zhang, 2015	27	215	62	213	16.6	0.43 [0.29, 0.65]
DANAMI 3, 2015	17	313	52	314	12.9	0.33 [0.19, 0.55]
PRAMI, 2013	16	234	46	231	12.5	0.34 [0.20, 0.59]
Politi, 2010	14	130	28	84	11.5	0.32 [0.18, 0.58]
Help−AMI, 2009	9	52	6	17	6.4	0.49 [0.20, 1.18]

RE Model for All Studies (Q = 12.54, df = 8, p for heterogeneity = 0.13; I^2 = 39.9%) — 0.33 [0.26, 0.42]

p for overall effect < 0.001

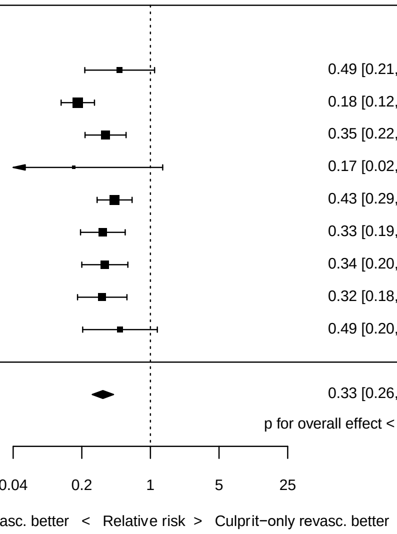

Figure S59. Sensitivity analysis for risk of unplanned revascularization excluding the DANAMI 3 trial

Study and Year	Active Events	Active N	Control Events	Control N	Weight (%)	Relative risk [95% CI]
Risk of unplanned revascularisation						
CvLPRIT, 2019	8	150	16	146	9	0.49 [0.21, 1.10]
Complete, 2019	29	2016	160	2025	14.7	0.18 [0.12, 0.27]
Compare ACUTE, 2017	18	295	103	590	13.4	0.35 [0.22, 0.57]
Hamza, 2016	1	50	6	50	2.4	0.17 [0.02, 1.33]
Zhang, 2015	27	215	62	213	14.4	0.43 [0.29, 0.65]
PRAMI, 2013	16	234	46	231	12.6	0.34 [0.20, 0.59]
Dambrink, 2012	27	79	15	40	13.1	0.91 [0.55, 1.51]
Politi, 2010	14	130	28	84	12	0.32 [0.18, 0.58]
Help−AMI, 2009	9	52	6	17	8.4	0.49 [0.20, 1.18]
RE Model for All Studies (Q = 27.20, df = 8, p for heterogeneity = 0.00; I^2 = 67.8%)						0.38 [0.27, 0.54]

p for overall effect < 0.001

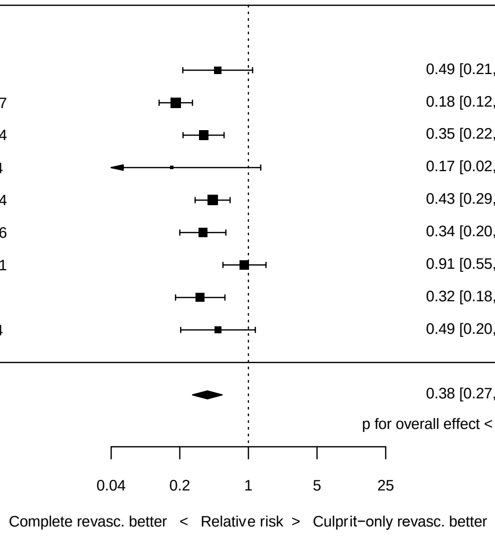

Figure S60. Sensitivity analysis for risk of unplanned revascularization excluding the Hamza trial

Study and Year	Active Events	Active N	Control Events	Control N	Weight (%)	Relative risk [95% CI]
Risk of unplanned revascularisation						
CvLPRIT, 2019	8	150	16	146	7.9	0.49 [0.21, 1.10]
Complete, 2019	29	2016	160	2025	13.6	0.18 [0.12, 0.27]
Compare ACUTE, 2017	18	295	103	590	12.2	0.35 [0.22, 0.57]
Zhang, 2015	27	215	62	213	13.3	0.43 [0.29, 0.65]
DANAMI 3, 2015	17	313	52	314	11.6	0.33 [0.19, 0.55]
PRAMI, 2013	16	234	46	231	11.4	0.34 [0.20, 0.59]
Dambrink, 2012	27	79	15	40	11.9	0.91 [0.55, 1.51]
Politi, 2010	14	130	28	84	10.8	0.32 [0.18, 0.58]
Help−AMI, 2009	9	52	6	17	7.3	0.49 [0.20, 1.18]
RE Model for All Studies (Q = 26.80, df = 8, p for heterogeneity = 0.00; I^2 = 67.6%)						0.38 [0.28, 0.52]

p for overall effect < 0.001

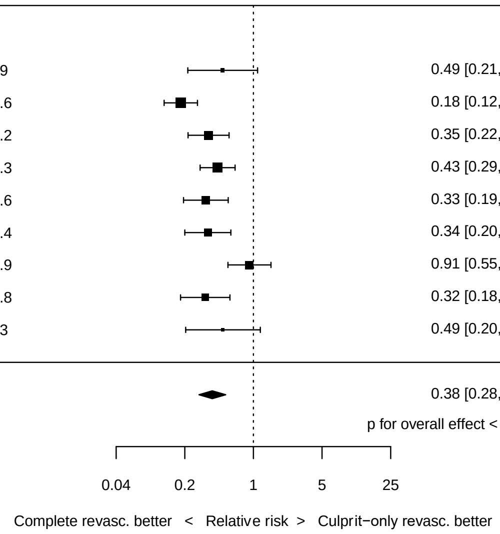

Figure S61. Sensitivity analysis for risk of unplanned revascularization excluding the HELP-AMI trial

Study and Year	Active Events	Active N	Control Events	Control N	Weight (%)	Relative risk [95% CI]
Risk of unplanned revascularisation						
CvLPRIT, 2019	8	150	16	146	8.5	0.49 [0.21, 1.10]
Complete, 2019	29	2016	160	2025	14.2	0.18 [0.12, 0.27]
Compare ACUTE, 2017	18	295	103	590	12.9	0.35 [0.22, 0.57]
Hamza, 2016	1	50	6	50	2.2	0.17 [0.02, 1.33]
Zhang, 2015	27	215	62	213	13.9	0.43 [0.29, 0.65]
DANAMI 3, 2015	17	313	52	314	12.2	0.33 [0.19, 0.55]
PRAMI, 2013	16	234	46	231	12	0.34 [0.20, 0.59]
Dambrink, 2012	27	79	15	40	12.6	0.91 [0.55, 1.51]
Politi, 2010	14	130	28	84	11.5	0.32 [0.18, 0.58]
RE Model for All Studies ($Q = 26.84$, df = 8, p for heterogeneity = 0.00; $I^2 = 68.2\%$)						0.37 [0.26, 0.51]

p for overall effect < 0.001

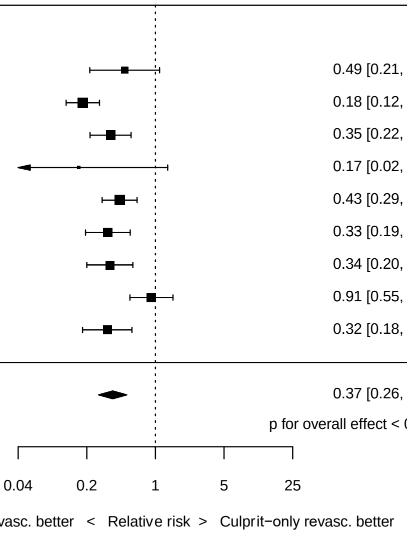

Figure S62. Sensitivity analysis for risk of unplanned revascularization excluding the Politi trial

Study and Year	Active Events	Active N	Control Events	Control N	Weight (%)	Relative risk [95% CI]
Risk of unplanned revascularisation						
CvLPRIT, 2019	8	150	16	146	8.9	0.49 [0.21, 1.10]
Complete, 2019	29	2016	160	2025	14.6	0.18 [0.12, 0.27]
Compare ACUTE, 2017	18	295	103	590	13.3	0.35 [0.22, 0.57]
Hamza, 2016	1	50	6	50	2.4	0.17 [0.02, 1.33]
Zhang, 2015	27	215	62	213	14.3	0.43 [0.29, 0.65]
DANAMI 3, 2015	17	313	52	314	12.7	0.33 [0.19, 0.55]
PRAMI, 2013	16	234	46	231	12.5	0.34 [0.20, 0.59]
Dambrink, 2012	27	79	15	40	13	0.91 [0.55, 1.51]
Help−AMI, 2009	9	52	6	17	8.3	0.49 [0.20, 1.18]
RE Model for All Studies (Q = 27.19, df = 8, p for heterogeneity = 0.00; I^2 = 68.1%)						0.38 [0.27, 0.54]
						p for overall effect < 0.001

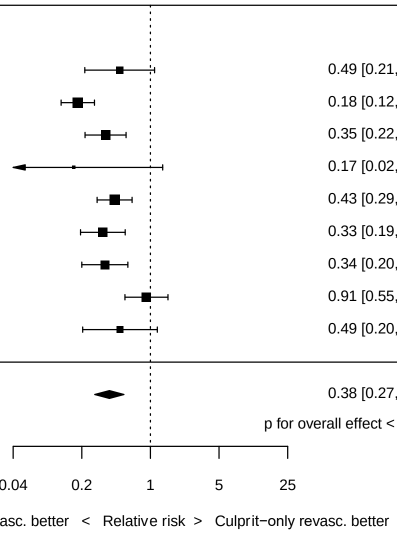

Figure S63. Sensitivity analysis for risk of unplanned revascularization excluding the PRAMI trial

Study and Year	Active Events	Active N	Control Events	Control N	Weight (%)	Relative risk [95% CI]
Risk of unplanned revascularisation						
CvLPRIT, 2019	8	150	16	146	9	0.49 [0.21, 1.10]
Complete, 2019	29	2016	160	2025	14.7	0.18 [0.12, 0.27]
Compare ACUTE, 2017	18	295	103	590	13.4	0.35 [0.22, 0.57]
Hamza, 2016	1	50	6	50	2.4	0.17 [0.02, 1.33]
Zhang, 2015	27	215	62	213	14.4	0.43 [0.29, 0.65]
DANAMI 3, 2015	17	313	52	314	12.8	0.33 [0.19, 0.55]
Dambrink, 2012	27	79	15	40	13.1	0.91 [0.55, 1.51]
Politi, 2010	14	130	28	84	12	0.32 [0.18, 0.58]
Help−AMI, 2009	9	52	6	17	8.4	0.49 [0.20, 1.18]
RE Model for All Studies (Q = 27.30, df = 8, p for heterogeneity = 0.00; I^2 = 68.1%)						0.38 [0.27, 0.54]

p for overall effect < 0.001

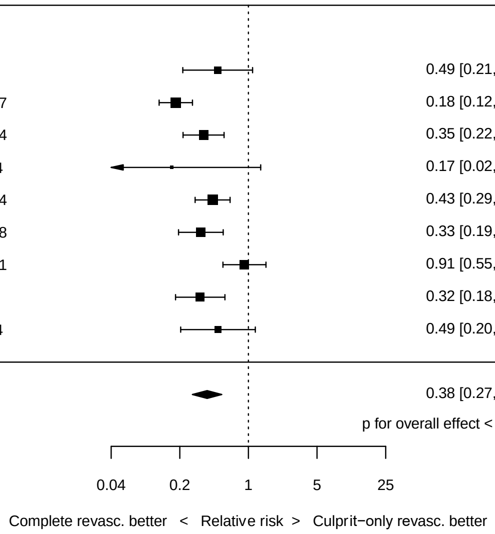

Figure S64. Sensitivity analysis for risk of unplanned revascularization excluding the Zhang trial

Study and Year	Active Events	Active N	Control Events	Control N	Weight (%)	Relative risk [95% CI]
Risk of unplanned revascularisation						
CvLPRIT, 2019	8	150	16	146	9.2	0.49 [0.21, 1.10]
Complete, 2019	29	2016	160	2025	14.9	0.18 [0.12, 0.27]
Compare ACUTE, 2017	18	295	103	590	13.6	0.35 [0.22, 0.57]
Hamza, 2016	1	50	6	50	2.4	0.17 [0.02, 1.33]
DANAMI 3, 2015	17	313	52	314	13	0.33 [0.19, 0.55]
PRAMI, 2013	16	234	46	231	12.8	0.34 [0.20, 0.59]
Dambrink, 2012	27	79	15	40	13.3	0.91 [0.55, 1.51]
Politi, 2010	14	130	28	84	12.2	0.32 [0.18, 0.58]
Help−AMI, 2009	9	52	6	17	8.5	0.49 [0.20, 1.18]
RE Model for All Studies ($Q = 26.42$, $df = 8$, p for heterogeneity = 0.00; $I^2 = 66.3\%$)						0.37 [0.26, 0.52]

p for overall effect < 0.001

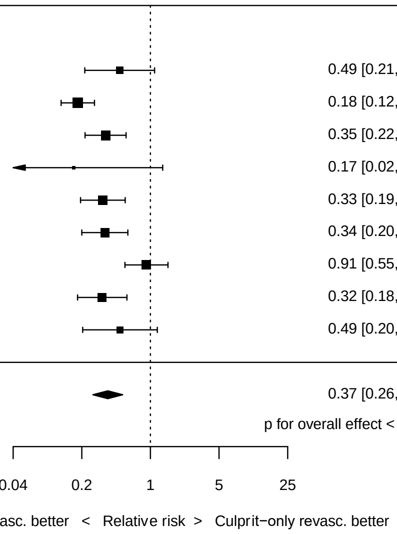

Supplement: Supplementary file 1 — Data S1 Table S1 Figures S1–S64 References 9, 16–24 [file JAH3-9-e015263-s001.pdf]
